# Mapping the structure and microstructure of the adolescent brain: Insights from the ABCD study^☆^

**DOI:** 10.1016/j.dcn.2026.101843

**Published:** 2026-10-30

**Authors:** Anthony Steven Dick, Donald J. Hagler, Alison Rigby, Mohammadreza Bayat, Marilyn Curtis, Raul Gonzalez, Carolina Makowski, Diliana Pecheva, Alexander L. Wallace, Richard Watts, Biplabendu Das, Janosch Linkersdoerfer, Olivier Celhay, Pravesh Parekh, Eva Müller-Oehring, Mustafa Okan Irfanoglu, Elizabeth A. Hoffman, Gayathri J. Dowling, Diana Alkire, Anders Dale, Terry Jernigan

**Affiliations:** a Department of Psychology and Center for Children and Families, Florida International University, Miami, FL, USA; b Center for Multimodal Imaging and Genetics, J. Craig Venter Institute, San Diego, CA, USA; c Department of Psychiatry, University of California, San Diego, La Jolla, CA, USA; d Center for Human Development, University of California, San Diego, La Jolla, CA, USA; e Department of Radiology, University of California, San Diego School of Medicine, La Jolla, CA, USA; f Department of Cognitive Science, University of California, San Diego, La Jolla, CA, USA; g Neurosciences Graduate Program, University of California, San Diego, La Jolla, CA, USA; h Department of Neuroscience, University of California, San Diego, La Jolla, CA, USA; i Faculty of Health, University of Canterbury, Christchurch, Canterbury, New Zealand; j National Institute of Biomedical Imaging and Bioengineering, National Institutes of Health, Bethesda, MD, USA; k Center for Health Sciences, SRI International, Menlo Park, CA, USA; l Department of Neurology and Neurological Sciences, Stanford University, Stanford, CA, USA; m Division of Extramural Research, National Institute on Drug Abuse, National Institutes of Health, Bethesda, MD, USA; n Centre for Precision Psychiatry, Division of Mental Health and Addiction, University of Oslo and Oslo University Hospital, Oslo, Norway; o University of Oslo, Oslo, Norway

**Keywords:** Adolescent Brain Cognitive Development (ABCD) study, Structural MRI, Diffusion MRI, Cortical morphology, Longitudinal neuroimaging

## Abstract

The Adolescent Brain Cognitive Development (ABCD) Study provides an unprecedented opportunity to characterize adolescent brain maturation and its correlates. This review synthesizes structural and diffusion magnetic resonance imaging (MRI) findings from ABCD, emphasizing methodological advances and implications for mental health, obesity, and environmental risk. We first summarize ABCD’s harmonized multi-site acquisition, high completion rates, and longitudinal coverage, highlighting the impact of quality control and scanner harmonization. Structural MRI work converges on largely linear cortical thinning from late childhood through early adolescence, surface area (SA) peaks in early adolescence followed by modest contraction, and regionally patterned but small associations with cognition, puberty, sex, and psychopathology. Genetic and twin analyses show that cortical thickness (CT) and SA are highly heritable yet partly dissociable, and that stable person-specific variance is not captured entirely by genetic influences. We then review diffusion imaging, focusing on Restriction Spectrum Imaging (RSI), which decomposes the diffusion signal into restricted, hindered, and free-water compartments and provides neurite- and cell-body-sensitive indices in both white and grey matter. ABCD RSI studies reveal widespread age-related increases in restricted diffusion in cortico–striatal–thalamic circuits and demonstrate that these microstructural signals are highly heritable in some brain regions. RSI also sharpens inferences about environmental and metabolic exposures: air pollution, central adiposity, and weight gain show associations with altered intracellular diffusion, particularly in reward circuitry. Finally, we describe FEMA, a fast mixed-effects framework that enables vertex- and voxelwise modeling at ABCD scale, and outline opportunities to integrate structural, diffusion, and genomic data into developmentally informed brain–behavior models.

## 1 Introduction

Adolescence is a critical period of neurodevelopment, characterized by rapid and dynamic changes in brain structure, microstructure, and connectivity. These changes support the refinement of cognitive abilities, socio-emotional processing, and self-regulatory capacities that are essential for adult functioning, while also coinciding with heightened vulnerability to the emergence of mental health disorders, substance use, and other adverse outcomes. Mapping the structural architecture of the adolescent brain, and understanding how it evolves over time, offers a unique opportunity to identify early markers of risk and resilience. The present synthesis draws on the remarkable scale, harmonization, and longitudinal nature of the Adolescent Brain Cognitive Development (ABCD) Study, distilling insights from its first waves of structural and diffusion magnetic resonance imaging (MRI) data. In doing so, it not only captures a snapshot of the literature throughout a pivotal developmental stage, but also gestures toward the broader arc of discovery that could emerge as these children enter adulthood, when the patterns, predictors, and mechanisms reviewed here may be re-examined in the context of mature brain organization and life outcomes.

The ABCD Study provides an unprecedented platform for this endeavor. Launched in 2016, ABCD is the largest longitudinal neuroimaging study of youth to date, following nearly 12,000 children recruited at ages 9–10 across 21 study sites in the United States, combining harmonized imaging with deep phenotyping and longitudinal follow-up (Casey et al., 2018). The scope, harmonization, and open-science ethos of ABCD have transformed developmental cognitive neuroscience by enabling reproducible insights into brain maturation, individual differences, and environmental influences at a national scale (Casey et al., 2018). This review focuses on structural MRI (sMRI) and diffusion MRI (dMRI), which are available across the entire cohort’s biennial visits.

sMRI provides high-resolution, quantitative measures of cortical thickness (CT), surface area (SA), regional and cortical volumes (CV) and other peri-cortical measures that have long served as primary indicators of developmental trajectories, including widespread cortical thinning with age, regional patterns of SA change, and subcortical volumetric reorganization across adolescence (Mills et al., 2016). However, sMRI alone provides limited insight into the underlying microstructural processes driving these changes, such as myelination, axonal packing, dendritic complexity, cell body density, and properties of the extracellular space.

dMRI complements and extends information offered by sMRI by probing tissue microstructure through water diffusion. Traditional Diffusion Tensor Imaging (DTI) metrics such as fractional anisotropy (FA) and mean diffusivity (MD) have allowed important insights into white matter maturation of known white matter pathways, but the single-tensor Gaussian assumption limits biological specificity and conflates effects across crossing fibers and multiple tissue compartments (Brunsing et al., 2017, Zhang et al., 2012). In contrast, multi-compartment diffusion models estimate signal contributions from restricted, hindered, and free water compartments, providing metrics that more directly relate to putative neurite density and orientation properties (Palmer et al., 2022).

Within ABCD, RSI has emerged as one informative framework. RSI decomposes the diffusion signal into restricted, hindered and free water compartments. The restricted compartment is further decomposed into components with directional and isotropic structure (often summarized as restricted normalized directional, RND, and restricted normalized isotropic, RNI). These measures provide more biologically interpretable indices than DTI, with greater sensitivity to microstructural features within both white and grey matter, and support large-scale, longitudinal inference in pediatric samples (Palmer et al., 2022, Watts et al., 2023). By incorporating a multi-shell diffusion protocol with extensive high b-value sampling (Hagler et al., 2019), the ABCD Study optimized its acquisition for multicompartment modeling across sites and waves, enabling more sensitive mapping of typical developmental trajectories and more mechanistically grounded brain–behavior associations of clinical relevance.

### 1.1 Overview of structural and diffusion imaging protocols

ABCD’s MRI protocols were centrally developed and harmonized across Siemens, GE, and Philips scanners to ensure reproducibility across 21 sites (Casey et al., 2018). High-resolution T1- and T2-weighted scans (1.0 mm isotropic) support surface-based morphometry (e.g., CT, SA, volume) and subcortical segmentation using standardized processing pipelines (Hagler et al., 2019). For structural data, preprocessing includes gradient nonlinearity correction and inhomogeneity correction, followed by FreeSurfer-based parcellation (Hagler et al., 2019, Desikan et al., 2006, Destrieux et al., 2010).

The dMRI protocol is a multi-shell diffusion acquisition with 1.7 mm isotropic voxels, seven b=0 volumes, and 96 diffusion-weighted directions distributed across four shells: 6 at b=500, 15 at b=1000, 15 at b=2000, and 60 at b=3000  s/mm^2^ (a total of 103 volumes including b=0). Siemens and GE sites collect these in a single acquisition, while Philips implements the identical scheme as two back-to-back runs that sum to the same counts. This design provides dense angular coverage at high diffusion weighting optimized for advanced compartment models such as RSI, which separates hindered and restricted diffusion signal at higher *b*-values. Acquisition blocks include reversed phase-encoding fieldmaps (blip-up/blip-down) for susceptibility correction. ABCD minimizes site effects by using vendor-matched acquisition protocols across scanner platforms and a uniform preprocessing pipeline (correcting gradient nonlinearity and intensity bias in sMRI; correcting motion, eddy currents, susceptibility distortions, and rotating the b-matrix in dMRI), with continuous phantom quality assurance to monitor scanner performance.

### 1.2 A note on heritability in ABCD

Heritability is the proportion of variance in a trait, such as CT, SA, or diffusion metrics, that can be statistically attributed to genetic differences among individuals in a given sample, as distinct from variance attributable to environmental factors (Visscher et al., 2008). It is a population-level statistic, not a fixed property of a trait or an individual. Two things follow from this. First, heritability can shift across development as genetic and environmental influences change in relative importance, and it can even reverse or vanish for a given trait depending on the age range and sample under study (Visscher et al., 2008). Second, the heritability of a trait’s baseline level and the heritability of its rate of change over time are not the same quantity and are often driven by different genetic variants (Brouwer et al., 2022). ABCD’s inclusion of twin and sibling pairs at select sites allows genetic and environmental contributions to be separated using classical twin modeling or, more recently, methods that incorporate genome-wide single nucleotide polymorphism (SNP) data directly. Several sections below report heritability estimates derived this way, along with related quantities such as genetic correlation (the degree to which the same genetic variants influence a trait across two timepoints or two regions) and the Fast and Efficient Mixed Effects Algorithm (FEMA)-derived A/C/S/E partition, which separates additive genetic (A), shared-environmental (C), stable person-specific (S), and residual (E) contributions to variance. Where these terms appear, we return briefly to what a given estimate does and does not imply about developmental mechanism.

### 1.3 Modeling site- and participant-related variance in structural imaging

Because residual site and scanner variance can remain after standard pre-processing steps (Pan et al., 2024), downstream analyses typically model site, scanner, or software release as covariates (Palmer et al., 2022), or apply retrospective harmonization (e.g., ComBat, CovBat, RELIEF, ABCD-Harmonizer procedures). Here, ComBat (Fortin et al., 2017) refers to an empirical-Bayes adjustment that removes site-specific mean/variance shifts while preserving prespecified covariate effects. CovBat extends ComBat to also align cross-feature covariance (Chen et al., 2022), and RELIEF is a multivariate approach that models and removes site-related mean and covariance to reduce site identifiability (Zhang et al., 2023).

Examinations of the effectiveness of these approaches, and comparisons across these practices, have been published using ABCD data, an ideal dataset for assessing the effects of vendor, site/scanner, and software differences. For example, Dudley et al. (2023) quantified scanner contributions in ABCD and found non-zero scanner-induced variance in every structural and diffusion MRI feature examined (e.g., CT, T2 intensity, mean diffusivity), with manufacturer and individual scanner often the dominant sources. Implementation of ComBat materially improved statistical power, particularly in smaller subsamples. Their ABCD-Harmonizer applies ComBat at the scanner-hash level (defining the batch), and for subsamples of N<250 yielded more accurate effect size estimates than modeling scanner as a covariate via ordinary least squares. However, the benefit diminished as sample size increased, and in large-N settings simply including scanner as a covariate produced comparable results. Thus, for many analyses the latter may be sufficient.

A natural question is how common feature-level harmonizers compare head-to-head in ABCD. Using ABCD baseline dMRI (FA, MD), Kraft et al. (2024) benchmarked ComBat, CovBat, and RELIEF with a site-classification test in which perfect harmonization drives area-under-the-curve (AUC) toward 0.50 (chance). In a controlled setting with equal per-site sample sizes drawn from large sites, all three methods reduced site identifiability and preserved age and sex signal, with RELIEF often achieving the lowest (best) AUC. In the full, unbalanced ABCD sample with many small-sample sites, RELIEF’s performance degraded and was more variable, improving only after stepwise removal of the smallest sites. ComBat and CovBat were less sensitive to this imbalance. Across-manufacturer comparisons also remained more challenging for all methods (AUCs higher than within-brand), indicating residual vendor signal even after harmonization.

In parallel, Cetin-Karayumak and colleagues (Cetin-Karayumak et al., 2024) released a voxel-level ABCD dMRI resource that harmonizes the raw diffusion signal across scanners. They learned a rotation-invariant spherical harmonics mapping to a common reference scanner and applied it to data from the remaining 32 scanners, reducing scanner differences and improving cross-site tractography consistency. This voxel-level harmonization addresses scanner effects at the signal stage, offering a complementary approach to dealing with cross-site effects.

In summary, ABCD’s prospective standardization reduces, but does not eliminate, site and vendor effects. For univariate feature analyses with large samples, modeling scanner/software as covariates may suffice, but for smaller or unbalanced samples, empirical-Bayes harmonization (e.g., ComBat) can recover power and reduce bias. Multivariate settings that depend on cross-feature structure (e.g., connectivity, tractography) may benefit from covariance-aware methods (CovBat, RELIEF) or signal-level harmonization applied to raw dMRIs. In all cases, best practice is to apply harmonization or model explicit scanner/software covariates, and potentially report manufacturer-stratified sensitivity analyses to verify effectiveness of the procedure.

Site and scanner are only one part of the modeling problem. ABCD’s scale makes even very small associations statistically detectable, with Pearson correlations as low as |r|≈0.04 detectable with 99% power at the full baseline sample size (Dick et al., 2021, Dick et al., 2025). This power is a strength, but it places the interpretive weight on effect sizes and on the choice of participant-level covariates rather than on statistical significance, since almost any association could reach significance. Several covariate choices recur throughout the findings we review below; we consolidate them here to give practical guidance and to avoid restating the same caveats at each mention. Following Dick et al. (2021), we emphasize that covariate selection should be principled and hypothesis-driven rather than reflexive. It is common practice to include covariates in an attempt to “purify” an association, but conditioning on a variable that shares a common cause with both predictor and outcome, or that lies on the causal path between them, can sometimes obscure a real effect rather than clarify it. The practical recommendation is therefore not to maximize or minimize covariates, but to specify each covariate’s hypothesized role, and to report models with and without it, comparing effect sizes and their confidence intervals rather than p-values (Dick et al., 2021).

With that principle in mind, the modeling of global brain size is the most consequential decision for structural phenotypes, because SA and CV scale with head size whereas CT is largely size-invariant. The choice among scaling specifications (e.g., intracranial volume, whole-brain volume, or total SA) therefore changes regional inferences and can create or erase apparent group differences. Marshall et al. (2025) showed that adjusting for whole-brain volume and total SA (rather than intracranial volume alone) yields the most stable interpretations and attenuates many apparent sex differences in SA and CV, while CT shows fewer robust sex effects regardless of specification. The same logic extends to psychopathology. Mewton et al. (2022) found that regional cortical volume and surface area associations with general psychopathology, externalizing, internalizing, and thought-disorder dimensions were widespread when modeled individually, but did not survive once global cortical volume and surface area were entered simultaneously, indicating that failure to model these global differences can produce apparently regional but in fact global and non-specific effects. Because no single scaling choice is correct for every question, we recommend reporting CT and SA separately, treating CV as a composite rather than a unitary measure, and documenting sensitivity to alternative scaling specifications.

Sociodemographic context is a second influential covariate, precisely because socioeconomic status (SES) and demographics share common causes with both brain structure and many outcomes of interest, which is the situation in which naive adjustment is most fraught. Palmer et al. (2021) illustrated both its magnitude and its structure. Adjusting for race/ethnicity, household income, and parental education reduced the out-of-sample variance in cognition explained by cortical regionalization roughly four- to eight-fold, and those variables accounted for 70%–90% of the cognitive variance that brain structure otherwise appeared to predict. More instructive is what adjustment did to the spatial patterns. Unadjusted, the vertex-wise association maps for fluid and crystallized performance were highly similar (r=0.69 for surface area, r=0.89 for thickness), but adjusted maps were largely distinct (r=0.30 and r=0.40, respectively). Unmodeled sociodemographic variance therefore may inflate effect sizes and impose a common spatial pattern across outcomes, one that is easily mistaken for a general neural substrate.

Rodriguez Rivera et al. (2025) similarly found that morphometric differences associated with early tobacco exposure were largely eliminated after adjustment for parental education, household income, and economic hardship. Whether SES should be treated as a confounder to adjust for, or as a substantive variable whose inclusion informs the model, depends on the research question (Dick et al., 2021). What matters is that the choice be explicit and that unadjusted associations be interpreted as upper bounds on the brain-specific effect.

In-scanner motion and data quality require comparable care, because motion is not random with respect to age or clinical status and can induce spurious developmental patterns. Ducharme et al. (2016) showed that retaining motion-affected scans produces artifactual quadratic or cubic age trajectories, whereas strict quality control and mixed-effects modeling recover predominantly linear thinning. Because ABCD’s true CT slopes are small, they are readily obscured or distorted when motion and quality control are not handled rigorously (Parsons et al., 2024). Finally, age, sex, and pubertal status interact, and inferences about any one depend on how the others are modeled. For example, pubertal status and tempo account for global variance in CT and SA beyond chronological age (Beck et al., 2023), and Marshall et al. (2025) showed that this moderation is also regionally patterned once brain-size scaling is handled correctly.

Taken together, these considerations explain why structural brain–behavior effect sizes in ABCD are small, and appropriately so. Well-powered analyses report typical brain–behavior correlations near r≈0.01, with upper-tail values rarely exceeding r≈0.16 (Marek et al., 2022), and effects of this magnitude are easily inflated by unmodeled global scaling, SES, or motion. A small effect is not necessarily an unimportant one, since it may still carry clinical or public-health relevance, be attenuated by measurement error, or accumulate across many factors (Dick et al., 2021, Dick et al., 2025). Throughout the sections that follow, we report effects with these caveats in mind rather than restating them at each instance, and we point the reader to more detailed treatments of effect-size interpretation and covariate selection in ABCD (Dick et al., 2021).

### 1.4 Completion metrics and implications

Imaging completion is very high at baseline and declines across follow-ups (Year 2, Year 4, Year 6). An important caveat is that, in the ABCD 6.0 data release, Year 6 only contains about half of the projected participants, so the completion rates at this timepoint are misleading. That said, the pattern of attrition is typical of longitudinal cohorts and substantially exacerbated in ABCD by COVID-19–related disruptions. As Fig. 1 shows, modality-specific differences are very small but consistent: T1 shows the highest completion, T2 is modestly lower, and dMRI is a little lower still, which may reflect the order of acquisition of the scans. At baseline, *overall* completion rates were 99.2% (T1), 95.7% (T2), and 95.2% (dMRI), which is comparable to other large pediatric cohorts (e.g., Generation R: 99% T1, 92% T2, 95% dMRI among 3992 children aged 9–11 years; White et al., 2018). By 2-year, 4-year, and 6-year follow-ups, *overall* completion declines to 68.4%, 53.6%, and 34.5% for T1; 67.1%, 52.8%, and 34.1% for T2; and 67.0%, 52.6%, and 34.0% for dMRI. These trends are broadly parallel across important demographic characteristics of sex, income, and ethnoracial groups: at Year 2, group-specific T1 completion ranges from roughly 59 to 73% across ethnoracial groups and 66 to 73% across income strata (with similar spreads for T2 and dMRI), and by Year 6 most groups fall in the mid-20s to high-30s. Across the later waves, White and higher-income participants tend to show somewhat higher completion than Black and lower-income groups, with Hispanic and Other groups intermediate; this pattern is consistent across T1, T2, and dMRI (see Supplementary Table 1). Some attention may be required to missing data for these variables, especially if they are central to the research question (Li et al., 2025).

Together, these structural and diffusion datasets enable integrated mapping of macro- and microstructure at scale, with sufficient power to properly examine competing developmental models, quantify within-subject change, and resolve small but reliable associations with cognition, mental health symptoms, obesity, and environmental exposures (Marshall et al., 2025, Palmer et al., 2022, Zhao et al., 2022a, Shen et al., 2021, Rapuano et al., 2022, Burnor et al., 2021). In the sections that follow, we (1) review contributions of sMRI and dMRI to characterizing typical development; (2) evaluate links to cognitive ability, mental health, substance use, obesity, and environmental exposures; (3) examine developmental correlates of puberty and sex differences; (4) summarize genetic and heritability findings from twin and genomic analyses; (5) highlight innovations and methodological challenges, particularly RSI and related multi-compartment models; and (6) outline opportunities to integrate structural, diffusion, functional, and genomic data to characterize individual neurodevelopmental profiles.

**Fig. 1:**
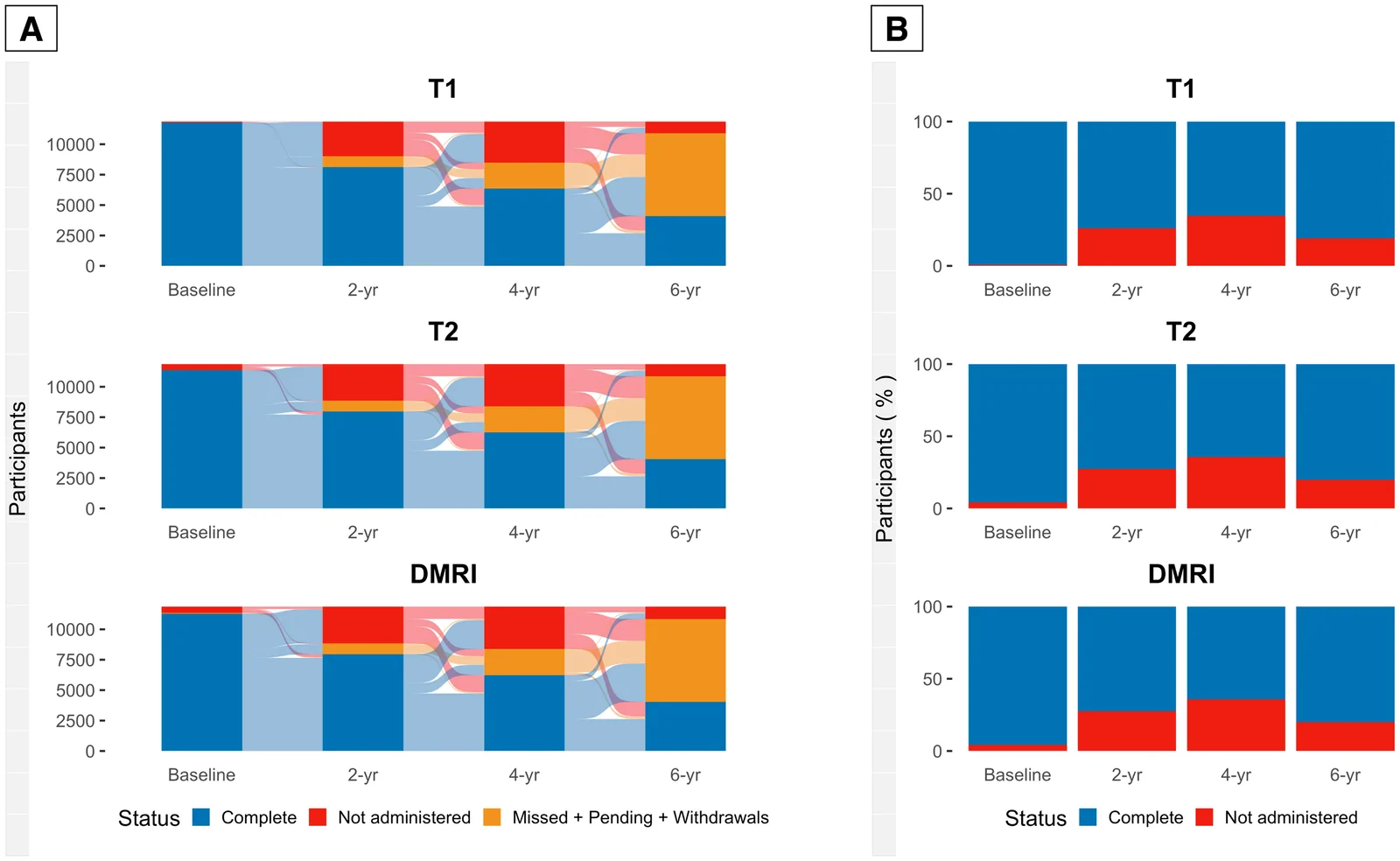
Completion rates for T1, T2, and dMRI scans. In A., the alluvial plot shows changes in group composition over the course of the study. Note the six-year pending rates are a sizeable percentage of scans because the study is ongoing. In B., the higher rate of “not administered” scans during the middle years is partially due to COVID-19 disruptions, which contributes to a subsequent rebound of completed scans in later years.

## 2 Typical brain development of cortical thickness, surface area, and volume

Adolescence is characterized by large-scale, regionally patterned changes in brain structure and tissue microstructure. Longitudinal and cross-sectional evidence from ABCD confirms and refines prior models of cortical and subcortical maturation.

**Fig. 2:**
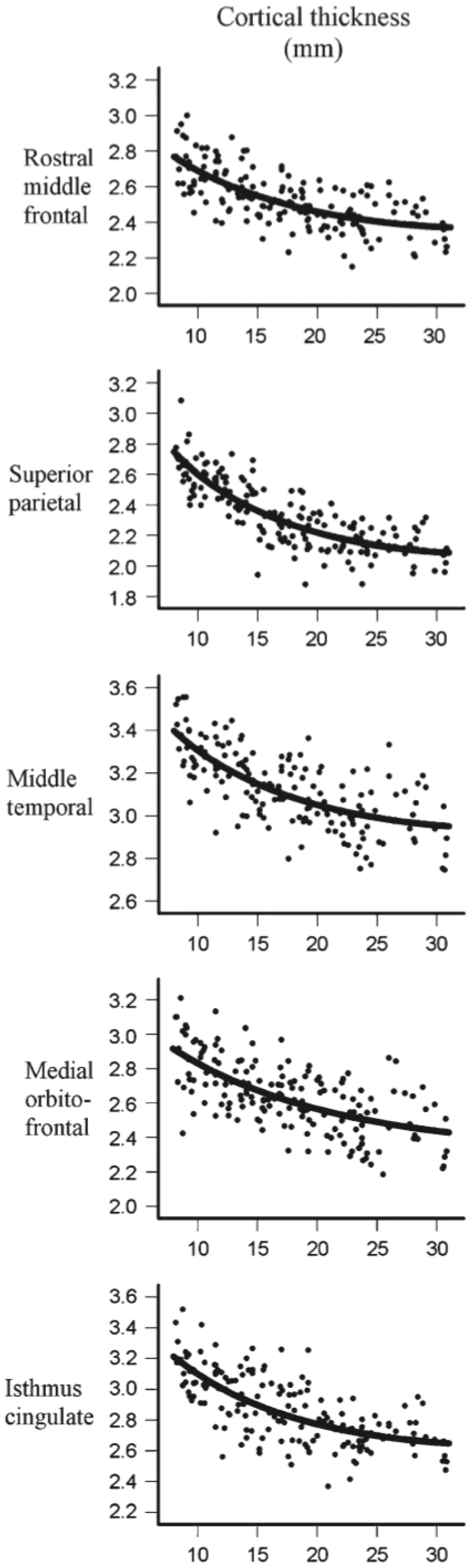
Age-related differences in cortical thickness across several regions of interest from cross-sectional data in 168 8-30-year-olds. Modified with permission from Tamnes, C.K., Østby, Y., Fjell, A.M., Westlye, L.T., Due-Tønnessen, P., Walhovd, K.B., 2010. Brain maturation in adolescence and young adulthood: regional age-related changes in cortical thickness and white matter volume and microstructure. *Cerebral Cortex, 20*, 534–548.

**Fig. 3:**
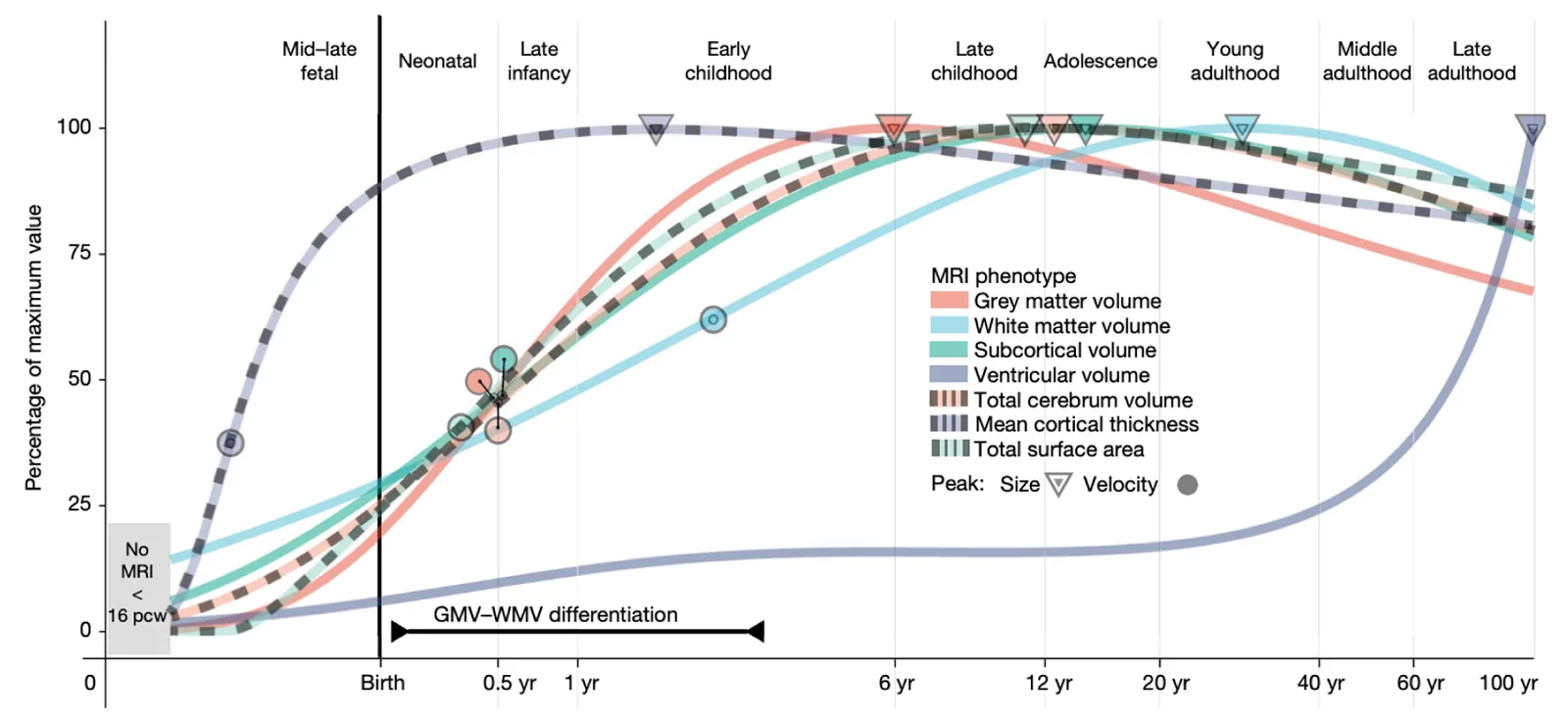
Lifespan trajectories of major structural MRI phenotypes from a large dataset comprising data from over 100,000 participants across public data. Substantial age-related differences are still apparent in adolescence. Reproduced from Bethlehem, R.A., Seidlitz, J., White, S.R., Vogel, J.W., Anderson, K.M., Adamson, C., Adler, S., Alexopoulos, G.S., Anagnostou, E., Areces-Gonzalez, A., et al. (2022). Brain charts for the human lifespan. *Nature, 604*, 525–533, under a CC BY 4.0 license.

### 2.1 What did we know before ABCD: Cortical thickness, surface area, and volume

Before the advent of large multi-site cohorts, early cross-sectional and mixed-longitudinal MRI studies converged on broad but regionally heterogeneous adolescent trajectories. Specifically, with appropriate control for total brain size, grey matter shows net reductions from childhood into adulthood while white matter increases more linearly into late adolescence, following a posterior-to-anterior gradient in which primary sensorimotor cortices mature before association regions and with lobe- and sex-specific nonlinear grey matter curves (e.g., Sowell et al., 2001, Giedd and Rapoport, 2010, Lenroot and Giedd, 2006, Gogtay et al., 2004, Lenroot et al., 2007). This early work also flagged that automated tissue classification and surface-based parcellation make inferences sensitive to motion, tissue-boundary contrast, and pipeline choices (Thompson et al., 2005). Importantly, in a single-sample, multimodal study (*n*=168; 8 to 30 years), Tamnes et al. (2010) found CT to be the most age-sensitive macrostructural marker, with white matter volume and DTI-derived changes only partly independent (see Fig. 2), suggesting that adolescent thinning reflects structural change in grey matter rather than merely the myelination of neighboring white matter. However, subsequent quantitative-MRI work shows that increasing myelination can shift the grey–white boundary and T1-weighted contrast, producing region-dependent “apparent” thinning (Natu et al., 2019, Lorio et al., 2016). The source of age-related differences in these indices is therefore not always clear within a single sample.

#### 2.1.1 Methodological refinements clarify cortical maturation trajectories

As pipelines matured, studies that explicitly separated CT and SA allowed more specific interpretation of developmental change. Wierenga et al. (2014) found the three measures dissociable in a single sample: average CT declined linearly, total SA followed a cubic trajectory, and CV was quadratic. Regional SA peaks tended to lag CV peaks, so some areas continued expanding laterally after volume had begun to decline. Mills et al. (2016) then addressed scaling directly across four longitudinal samples, showing that intracranial and whole-brain volume follow distinct trajectories and are not interchangeable, and that the choice of global correction alters both the shape of the modeled trajectory and whether sex effects emerge at all. Together with the quality-control evidence of Ducharme et al. (2016), this work establishes that CV behaves as a composite of dissociable CT and SA trajectories, and that apparent developmental “inflections” are often artifacts of unmodeled size, motion, or the conflation of CT and SA.

Controlling for artifacts, scaling, and site effects aligns longitudinal and cross-sectional findings. Thus, adolescence shows linear thinning, SA peaks in early adolescence followed by modest contraction, and declining CV. Longitudinal cohorts such as NCANDA (Brown et al., 2015) and cross-sectional samples such as PING show similar age effects but differ in slope, highlighting design influences (Pfefferbaum et al., 2016), while harmonization efforts and lifespan meta-analyses (LIFESPAN, ENIGMA, Brain Charts) confirm that modeling site effects and applying strict quality control recovers steep adolescent thinning and region-specific SA peaks (Pomponio et al., 2020, Frangou et al., 2022, Bethlehem et al., 2022; see Fig. 3).

#### 2.1.2 Genetic context

Twin and family designs established that macrostructural indices are highly heritable and partly dissociable, with distinct genetic influences on SA versus CT (Panizzon et al., 2009) and a caution that phenotype choice matters for inference (Winkler et al., 2010). CT heritability increases from childhood into adolescence (Schmitt et al., 2014), and both traits show heritable variation from the neonatal period onward (Jha et al., 2018). Their genetic architectures are nonetheless largely distinct, with the strongest genetic influences on CT and SA localizing to different cortical regions (Joshi et al., 2011, Yoon et al., 2012, Strike et al., 2019, Schmitt et al., 2019). The large ENIGMA genome-wide association study (GWAS) confirms that SA-associated variants cluster in regulatory regions active during mid-fetal corticogenesis (arealization and progenitor proliferation), whereas CT signals are stronger for postnatal processes such as synaptic organization and remodeling (Grasby et al., 2020). Intracortical myelin is also moderately heritable but only partially overlaps genetically with CT and SA (Schmitt et al., 2020). Conceptually, this confirms that CV is not a unitary construct: it conflates two traits with different trajectories, scaling properties, and genetic underpinnings, so wherever possible CT and SA should be modeled separately.

#### 2.1.3 Where the field has landed: Three patterns and a caution

Integrating the early mapping literature with rigorous longitudinal modeling and harmonized large-samples yields a stable consensus: (1) CT declines broadly and near-monotonically from late childhood through adolescence, with the steepest change from late childhood into mid-adolescence and few regional deviations; (2) SA follows an inverted-U, expanding from childhood to an early-adolescent peak and declining thereafter, with regional heterogeneity and sensitivity to scaling choices; and (3) CV, being a composite of these measures, declines from mid-childhood onward, initially because thinning outpaces continued SA expansion, and more steeply once SA passes its peak and begins to contract (e.g., Bethlehem et al., 2022, Frangou et al., 2022). The accompanying caution is methodological. Many sex and group differences in SA and CV diminish after appropriate head-size scaling, motion correction, and quality control, whereas cortical thinning is less affected, so failure to model these factors can qualitatively alter inferred developmental functions.

These established regularities came with methodological lessons of strict motion and quality control, appropriate brain-size scaling, and cross-site harmonization. Together they set the stage for ABCD’s contribution, which is the precise estimation of developmental slopes in early adolescence, explicit modeling of site and software variance and metric reliability, and refined tests of sex, puberty, and contextual effects. With its scale, harmonized acquisition/processing, and longitudinal depth, ABCD is positioned to adjudicate lingering cross-sectional vs. longitudinal tensions and address these questions head on. In the next section, we examine what we have learned about CT, SA, and CV development so far from ABCD, and delineate remaining questions that can be addressed in the coming years.

### 2.2 What we have learned from ABCD: Cortical thickness, surface area, and volume

ABCD’s harmonized acquisition and processing provide a stringent test of adolescent macrostructural trajectories in a large, demographically diverse cohort (Casey et al., 2018, Hagler et al., 2019). It is only recently that longitudinal data across more than two timepoints have become available for analysis, so most published longitudinal ABCD studies have examined two timepoints. For example, Marshall et al. (2025) examined 6323 children, aged 9 to 13 years, who had two scans (baseline and 2-year-follow-up), and reported widespread cortical thinning and widespread volume decline, the two largest age effects among the four metrics examined. SA change over this interval was smaller in magnitude and regionally mixed in direction, with frontotemporal and medial-occipital increases alongside parietotemporal decreases. Notably, the modal individual-level pattern was thinning paired with expansion rather than contraction (39.3% of region-by-participant observations in males, 37.6% in females).

Studies using ABCD data also quantify measurement properties that influence the interpretation of reported developmental slopes. Longitudinal stability varies across regions and metrics. Parsons et al. (2024) found that the CT intraclass correlation (ICC) mean was 0.76 (0.54 to 0.90), SA was 0.93 (0.82 to 0.97), and CV was 0.93 (0.76 to 0.97). Thus, CT generally proved less stable than SA or CV, with nontrivial site-level variance. These characteristics underscore the need to model site and reliability explicitly (e.g., manufacturer and/or software covariates) when estimating developmental change and group effects (Parsons et al., 2024). Together, these results suggest that in early adolescence CT is the most age-sensitive macrostructural signal, but also the noisiest. In contrast, SA and CV are more stably measured, though CV is itself strongly age-sensitive; it is SA that changes least over the two-year window, and in regionally mixed directions. A practical implication for ABCD is that small but real CT slopes can be obscured or distorted if quality control is not handled rigorously.

#### 2.2.1 Cognition, puberty, and sex differences

Cross-sectional ABCD analyses relating CT and SA to cognition, with varying adjustment for age, sex, site or scanner, and head size, report reliable but small associations (Zhao et al., 2022a, Acosta-Rodriguez et al., 2024, Patel et al., 2022). Consistent with the effect-size expectations set out in Section 1.3, these associations generally require large samples for stable detection (Marek et al., 2022). As we also discussed in Section 1.3, sociodemographic adjustment substantially reduces the brain–cognition associations that structural measures otherwise appear to explain (Palmer et al., 2021), a caveat directly relevant to interpreting multimodal models. However, recent work by Michel et al. (2024) suggests that integrating multimodal data can yield stronger associations. By employing regularized structural equation modeling to capture the complementary roles of grey and white matter, they were able to explain 19.0% of the variance in cognitive performance, which is higher than models using only grey matter (15.4%) or white matter (12.4%) alone. Importantly, however, unlike Palmer et al. (2021), these models in Michel et al. (2024) did not control for SES. Given Palmer et al.’s finding that sociodemographic factors can account for a large proportion of the shared variance between brain structure and cognition, this 19.0% estimate likely includes substantial variance attributable to environmental confounds. Furthermore, contrary to the common focus on CT, and converging with the cross-sectional pattern noted above, Michel et al. (2024) found that SA and CV were stronger predictors of cognition than CT, while white matter volume outperformed metrics from the DTI model. Thus, while individual structural metrics often show small effects in cross-sectional analyses, combining modalities and accounting for specific regional configurations, albeit potentially confounded by environmental factors, may provide a more comprehensive view of the brain–cognition relationship.

Given that cross-sectional structural metrics explain limited unique variance in cognition, often correlated with environmental factors, recent work has focused on isolating the specific biological mechanisms driving structural change. In ABCD, pubertal status and sex account for variance in global CT and SA beyond chronological age. Beck et al. (2023) tested temporal coupling between caregiver-reported Pubertal Development Scale (PDS) scores and whole-brain MRI metrics (global CT, SA, FA, MD) in 8896 baseline participants (mean age 9.9 y) and 6099 at two-year follow-up (mean age 11.9 y), adjusting for age, BMI, SES, and genetic-ancestry covariates and harmonizing site with longitudinal ComBat. Baseline pubertal status predicted subsequent cortical thinning in females (β=−0.08, p<.01) but not males, though this sex difference only marginally survived FDR correction ($p_{corr}=.049$). Change in PDS and change in CT were likewise negatively correlated in females (r=−0.03). For SA, the coupling path from baseline PDS to subsequent change was negative in both sexes (females β=−0.07; males β=−0.03; both p<.01), significantly stronger in females ($p_{corr}<.01$). Males alone showed two further bivariate latent change score (BLCS) parameters: baseline PDS correlated with concurrent SA (r=0.04) and change in PDS with change in SA (r=0.02). Together, these results indicate that, over and above age, pubertal status explains variance in global cortical maturation, with the clearest coupling for CT in females and for SA in both sexes, albeit with small effect sizes.

Complementing this emphasis on biological drivers of change, Marshall et al. (2025) show that apparent sex differences in ABCD depend strongly on how global brain size is modeled. With the better-fitting covariates, females showed steeper thinning and volume decline across much of the cortex, while sex differences in SA change were minimal (see Fig. 4). With a worse-fitting covariate (ICV), SA models would have wrongly suggested greater SA decline in females. Annual-percentage-change analyses also suggest a difference in timing rather than magnitude. In both sexes, thinning with expansion gave way to thinning with contraction, but in several regions this shift appeared about a year later in males. This may reflect sex differences in pubertal timing, but two-wave data cannot separate age from puberty or pin down the size of the lag, so additional waves are needed.

Taken together, Beck’s pubertal coupling and Marshall’s covariate-sensitivity results suggest that puberty and sex modulate the timing of early-adolescent CT and SA change beyond age, and, in Marshall’s regional analyses, its spatial patterning, with small effects.

**Fig. 4:**
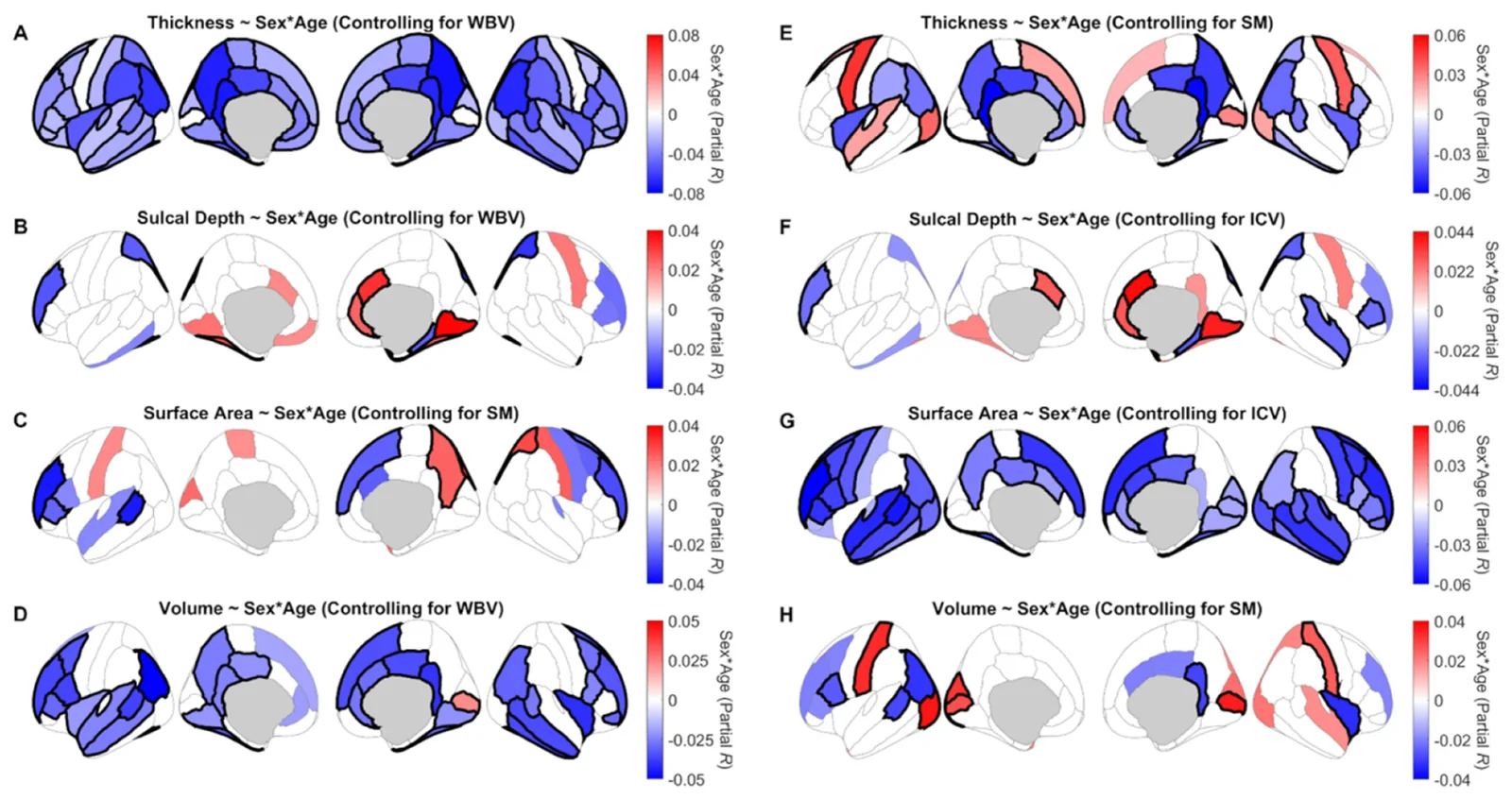
Sex by age interactions on brain structure, controlling for different whole–brain covariates. Panels show that the pattern of moderation by sex depends on what covariates are controlled in the model. Reproduced from Marshall, A.T., Adise, S., Kan, E.C., Sowell, E.R., 2025. Longitudinal sex- at-birth and age analyses of cortical structure in the ABCD study. *J Neurosci 45*(10):e1091242025, under a CC BY 4.0 license.

#### 2.2.2 Genetics and heritability of structural brain development in ABCD

As reviewed earlier, twin/family and genomic studies show that cortical SA and CT are distinct, highly heritable traits, with CT heritability rising through adolescence (Panizzon et al., 2009, Schmitt et al., 2014). Large GWAS meta-analyses suggest different developmental biology for each. Thus, genetic signals for SA point to prenatal processes that shape the layout and size of cortical regions, whereas CT-related signals point more to postnatal processes like synaptic remodeling (Grasby et al., 2020). With that backdrop, ABCD adds fine-grained, vertex-level maps and modeling that explicitly accounts for scanner and test–retest stability, allowing these genetic influences to be tested during early adolescence in a large longitudinal sample.

ABCD “moves the needle” in two concrete ways. First, it maps genetic effects at finer spatial resolution, and second, it illustrates the separation of stable person-specific variance from heritability in a large, harmonized pediatric sample. Using ABCD, Smith et al. (2024) partitioned vertex-wise variance in cortical measures into A/C/S/E (A additive genetic; C = shared family; S = stable subject-specific variance across waves; E = residual), adjusting for age, sex, scanner, and software. They found small C, substantial A for both CT and SA (with SA generally higher), and a sizeable S component. Stated as proportion of residualized variance ([0,1] scale), they find that, when averaged within ROIs, A(CT) ranged from 0.19 (temporal pole) to 0.44 (cuneus), while A(SA) was consistently higher, ranging from 0.28 (entorhinal cortex) to 0.57 (pericalcarine cortex). S is prominent over cingulate/precuneus/parietal regions for both CT and SA. For sulcal depth, S ranged from 0.34 (temporal pole) to 0.74 (caudal middle frontal gyrus) at the ROI level, while A was modest across most of the cortex. This shows that strong within-person stability does not automatically imply high heritability. Vertex-level maps also reveal that genetic contributions vary continuously within classical ROIs, so anatomical parcels do not necessarily align with “genetic parcels”.

Building on this distinction between anatomical and genetic boundaries, Makowski et al. (2022) demonstrated that using genetically informed atlas parcellations derived specifically from genetic correlations rather than sulcal/gyral patterns significantly enhances the discovery of genomic loci. In a massive GWAS combining the UK Biobank (discovery) and ABCD (generalization) datasets, they identified 440 genome-wide significant loci associated with cortical morphology. Crucially, they found that the genetic architecture of the cortex is largely generalizable from childhood to adulthood, particularly for surface area. Thus, genetic effects in the ABCD sample were strongly correlated with those in the adult UK Biobank, particularly for surface area (r range: 0.46 to 0.92). Beyond increasing discoverability, this approach revealed that cortical phenotypes are under strong negative selection and are enriched for regulatory elements in neurodevelopmental pathways. They further identified specific inversion polymorphisms, such as 17q21.31, that exert opposing effects on surface area and thickness, offering a genetic mechanism for the negative association between area and thickness often observed in biostatistical models of brain size.

Conceptually, the S term is the stable, person-specific part of the signal across time (after accounting for age, sex, scanner, and software), i.e., it is not the same as genetic influence. This helps explain why some regions look very stable across waves even when heritability (A) is only moderate, and why ROI averages can hide sharp within-ROI differences in both A and S. Set against the pre-ABCD literature, ABCD reinforces the SA–CT genetic dissociation and adds pediatric, multi-site, and scanner-adjusted estimates that make the spatial patterning more clear, and the interpretation less sensitive to data-quality differences.

A complementary line of evidence connects genetic propensity to cortical morphology while emphasizing global scaling and environment. In an independent adolescent cohort, Judd et al. (2020) showed that a polygenic index for educational attainment (EduYears-PGS) relates mainly to global SA. But once total SA is controlled, the remaining regional signal is restricted to right intraparietal sulcus. By contrast, SES shows broad global effects on SA at baseline and uniquely predicts change in total SA from ages 14 to 19, while EduYears-PGS does not. Together with ABCD’s variance partition, the picture is that (1) genetic influences on CT and SA are real but spatially heterogeneous and often small at any single location; (2) environmental/SES influences are large and global for SA and matter for longitudinal change; and (3) modeling global scaling is essential to avoid mistaking global effects for local ones. In short, ABCD does not overturn the pre-ABCD genetics. Rather, it solidifies the general conclusions, but adds vertex-level specificity. Furthermore, as a statistical testbed, ABCD shows promise for cleanly separating stable person-specific signal from heritability.

#### 2.2.3 Substance use and abuse

In current ABCD releases, adolescent substance use is only beginning to emerge (Sullivan et al., 2022); consequently, few studies have reported longitudinal morphometric change as a function of exposure. Even so, recent ABCD papers clarify and sometimes qualify associations reported in earlier, non-ABCD cohorts. Dai et al. (2022) reported that preadolescents who had ever used tobacco or nicotine by ages 9 to 10 showed lower crystallized cognition scores and slightly smaller total cortical SA and volume, with regionally reduced area and volume across frontal, parietal, and temporal cortex at baseline and 2-year follow-up. However, Rodriguez Rivera et al. (2025) demonstrated that many of these cognitive and morphometric differences were eliminated or substantially attenuated after adjustment for socioeconomic factors (parental education, household income, and economic hardship) and in propensity-matched subsamples, underscoring that early tobacco exposure is tightly intertwined with SES-related disadvantage rather than representing a clean exposure effect. Against this backdrop, Miller et al. (2024) tested whether baseline (ages 9 to 11) imaging predicts self-reported early initiation of alcohol, nicotine, or cannabis (before age 15). Early initiators showed small but widespread differences in larger global volumes (whole brain, cortical, and subcortical) and greater cortical SA (β≈0.04–0.05). CT effects were regionally patterned, with thinner prefrontal cortex (e.g., right rostral middle frontal) but thicker temporal, occipital, and parietal cortex (see Fig. 5). Alcohol largely recapitulated this topology and additionally showed thinner bilateral superior frontal and thicker bilateral parahippocampal cortex. Substance-specific findings included lower right caudate volume for cannabis and lower right superior frontal volume plus deeper left lateral orbitofrontal cortex for nicotine. Critically, when analyses were restricted to youth who initiated after baseline, most global and several regional associations persisted and were robust to prenatal-exposure adjustment, suggesting that some structural features precede use and may index predispositional risk rather than (or before) exposure effects. Effect sizes were uniformly small (typical β≈0.03–0.05). This is in alignment with other ABCD studies predicting substance use onset, which also note the importance of other risk factors above neuroanatomical markers (Green et al., 2024). Practically, these should be viewed as low-magnitude risk markers rather than diagnostic signatures, motivating longitudinal, within-person analyses to test onset, dose–response, and potential recovery.

Complementing these phenotypic results, Kramer et al. (2025) used the ABCD twin/sibling cohort at baseline (ages 9 to 11; drug-naïve) to ask whether polygenic risk for substance involvement relates to brain morphology *prior* to exposure. In multivariate ACE models including both ROI morphometry polygenic risk scores (PRSs) and substance PRSs, the cannabis use disorder PRS showed no reliable associations with any of eight ROIs (six subcortical volumes, total cortical SA, mean CT). A general SU/SUD PRS showed only very small links to nucleus accumbens volume and total cortical SA (standardized paths β≈0.02–0.06; ∼0.2% to 0.4% variance explained), whereas ROI-specific brain PRSs predicted their matched ROIs more strongly yet still modestly (∼0.8% to 4.4%). Twin modeling indicated predominantly additive-genetic influences on these morphometric traits with limited shared-environment effects. Kramer et al. (2025) explicitly reconciled these null genetic findings with the phenotypic associations reported by Miller et al. (2024) by suggesting that the structural differences observed in initiators, while present prior to use, may not be driven by the specific addiction genetic liabilities captured by current PRSs. Instead, they propose that the associations observed by Miller et al. might reflect environmental confounding that phenotypic designs cannot fully control, indirect genetic pathways not specific to SUD risk, or that definitive neuroanatomical predispositions simply have not yet developmentally emerged in the 9 to 11 age range. Taken together with non-ABCD longitudinal work such as Albaugh et al. (2021), which reported dose-dependent, accelerated prefrontal thinning among adolescents who used cannabis, these near-null genetic associations in drug-naïve ABCD youth suggest that many substance-related anatomical differences either reflect exposure or emerge later in development, rather than indexing strong pre-exposure genetic liability in gross anatomy at ages 9 to 11. As ABCD accrues additional waves, longitudinal models that estimate within-person slopes (e.g., for CT and SA), align effects to timing of first use, and test PRS-by-time and use-by-time interactions (ideally with twin/sibling contrasts and negative-control analyses) will be essential to adjudicate predisposition versus consequence and to detect any subtle, region-specific exposure effects.

**Fig. 5:**
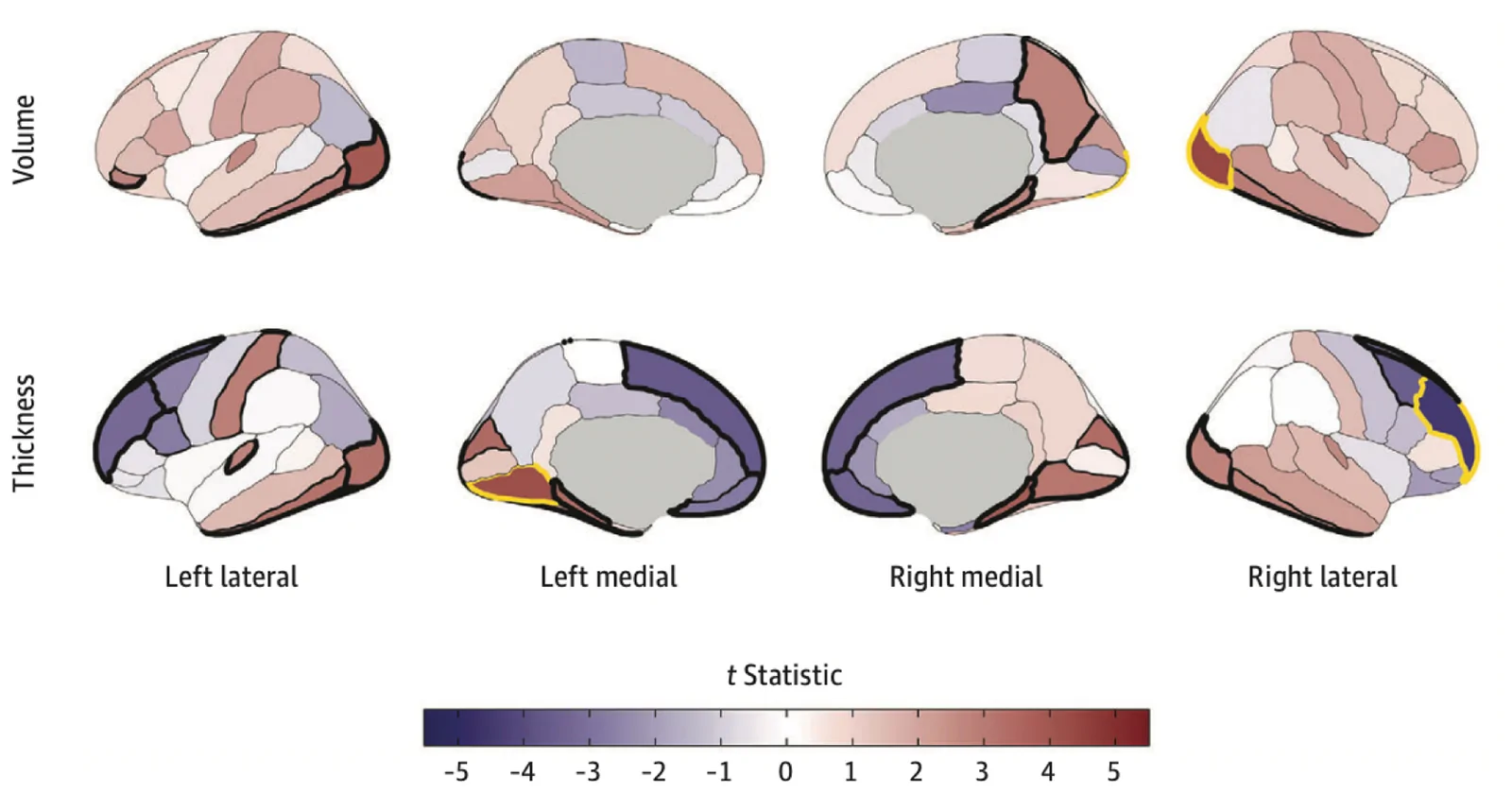
Regional Cortical Volume and Thickness Associations With Early Substance Use Initiation in the Adolescent Brain Cognitive Development (ABCD) Study. Cortical volume and thickness patterning of associations with any substance use initiation are plotted as *t* statistics (red indicates a positive association and blue indicates a negative association). Reproduced from Miller, A. P., Baranger, D. A. A., Paul, S. E., Garavan, H., Mackey, S., Tapert, S. F., LeBlanc, K. H., Agrawal, A., and Bogdan, R. (2024). Neuroanatomical variability and substance use initiation in late childhood and early adolescence. *JAMA Network Open, 7*(12), e2452027, under a CC BY 4.0 license.

#### 2.2.4 Mental health outcomes

Adolescent internalizing symptoms show small but consistent associations with brain morphology in ABCD. For example, Shen et al. (2021) reported that higher depressive symptoms and diagnosis were associated with lower global cortical volume and total SA, alongside regional volume and area reductions concentrated in frontal, temporal, and parietal cortex and reduced global FA (β = -.019 to -.057; Cohen’s *d* = -.018 to -.041). CT showed no association at either the global or regional level. These regional effects were carried almost entirely by caregiver-reported symptoms, with child self-report yielding a single significant regional association. The overall pattern reinforces a distributed (rather than focal) structural profile for depression in youth. Extending to transdiagnostic dimensions, Patel et al. (2022) examined the general psychopathology factor alongside cognition and found that surface area showed the most consistent associations with both, while CT showed little relation to psychopathology, with effects estimated adjusting for age, sex, ethnicity, and scanner, again emphasizing effect sizes that are reliable at scale but modest per region. Together these findings suggest that internalizing liability in late childhood is reflected by diffuse, small differences across much of the cortex rather than large regional shifts.

Socioeconomic context is a critical lens for interpreting internalizing–structure links in ABCD, as discussed in Section 1.3. Poverty is associated with lower cortical surface area and volume across multiple frontal, temporal, and occipital regions, with no cortical thickness clusters surviving correction (Kim et al., 2022). Notably, in that study poverty predicted increases in externalizing but not internalizing problems over one year, so its relevance here is primarily to how global and sociodemographic variation is modeled rather than to internalizing symptoms specifically. This clarifies cross-study discrepancies prior to ABCD, since differences in how SES and global scaling were treated can inflate or attenuate regional findings.

Within anxiety-related phenotypes, ABCD studies point to small, regionally specific volumetric differences rather than a single shared profile. Hammoud et al. (2024) found largely non-overlapping correlates for current anxiety disorders and behavioral inhibition. Lower behavioral inhibition tracked larger cerebral white matter and thalamic volume, whereas lower odds of a current anxiety disorder tracked larger total cortical grey matter, amygdala, and precentral gyrus volume. Dimensional models of internalizing show effects that are reliable at scale but sensitive to covariate specification, attenuating once cognitive performance is modeled, with regional cortical volume associations no longer significant while global associations persisted (Mewton et al., 2022).

Externalizing and related disinhibitory traits show a similar pattern of small, globally distributed associations. Mewton et al. (2022) modeled general psychopathology alongside its externalizing, internalizing, and thought-disorder subfactors, and found that higher scores on every dimension were associated with lower global cortical volume and surface area, with no association to cortical thickness anywhere. Regional effects were widespread but did not persist once global volume and surface area were modeled. No unique associations survived when all three were entered simultaneously, indicating that the effect is global and driven by shared rather than dimension-specific variance.

ABCD studies that integrate behavior or environment with brain structure reinforce this distributed picture for externalizing. Zhao et al. (2022b) related screen-media use and externalizing behaviors to a thalamus–prefrontal–brainstem structural covariation component (β = 0.107 and β = 0.117, respectively), a pattern that replicated closely against an independent adult sample. Complementing this, Nakua et al. (2025) examined cross-sectional and longitudinal links between distinct dimensions of externalizing and cortical thickness and subcortical volume in *a priori* frontolimbic and striatal regions, finding small associations that varied by dimension and, longitudinally, that higher baseline CT in left *pars triangularis* and rostral middle frontal gyrus predicted greater decreases in externalizing symptoms over two years. Together, these results indicate that externalizing risk in ABCD is detectable structurally, but only as subtle, network-distributed differences once key confounds are handled.

Finally, ABCD clarifies transdiagnostic structure–psychopathology relations. Studies modeling a general “*p*-factor” consistently find the strongest and most reproducible associations with global morphometric measures, especially total cortical SA and cortical volume (Romer et al., 2023), though the same study found that lower baseline CT predicted steeper within-person declines in internalizing symptoms, an effect that survived adjustment for global CT and for the *p*-factor. Modeling symptom dimensions separately rather than a general factor, Vargas and Mittal (2023) likewise identified regional CT and SA predictors that were partly shared across psychosis-like, depressive, and anxiety symptoms (e.g., superior frontal) and partly dimension-specific. These effects typically shrink (but remain directionally consistent) with rigorous control for SES, site or scanner, and global size, aligning with the broader lesson that adolescent psychopathology maps onto many tiny structural differences rather than any single anatomical marker. Building on this, newer ABCD work continues to evaluate diagnostic contrasts using longitudinal designs. Chen et al. (2025) compared propensity-matched lifetime MDD cases and controls and found group by time differences in cortical volume across frontal, temporal, and occipital regions, indicating divergent developmental trajectories rather than a single case–control offset. As longitudinal ABCD waves accrue, designs that estimate within-person slopes, align analyses to symptom onset and recovery, and incorporate twin and sibling contrasts will be pivotal for adjudicating trait-like liability versus state-linked change across internalizing, externalizing, and transdiagnostic dimensions.

#### 2.2.5 Summary

Across cognition, puberty, substance use, and psychopathology, ABCD structural findings share a characteristic magnitude and spatial extent. Associations are reliable but small, and spatially distributed rather than focal, often to the point that regional effects do not survive adjustment for global measures. Cortical thinning is the most age-sensitive macrostructural signal but also the least reliably measured, and where CT relates to symptoms its direction varies by dimension and region rather than pointing one way. SA and CV are more stably measured, though SA changes least over the two-timepoint window that characterizes most current ABCD analyses, and in regionally mixed directions. For cognition, SA and CV outperform CT as predictors, and sociodemographic adjustment substantially reduces the shared variance. Puberty and sex primarily shift the timing and regional focus of CT and SA change rather than its overall magnitude. For substance use, ABCD shows that some anatomical differences in early initiators precede first use, though they are small and poorly captured by current polygenic indices in drug-naïve youth; where exposure-based differences have been re-examined under sociodemographic adjustment, many attenuate substantially. For psychopathology, effects are largely global and shared across dimensions rather than diagnosis-specific. Genetic analyses reinforce the CT/SA dissociation while showing that stable person-specific variance is not reducible to heritability. Throughout, the apparent size of these effects depends heavily on how global brain size, sociodemographic context, and site or scanner are modeled, so the covariate considerations of Section 1.3 are essential to interpreting any single result.

## 3 Typical development of white and grey matter microstructure

Since the advent of clinically useful diffusion pulse sequences in the 1990s, diffusion–weighted imaging has become a primary tool to characterize brain development from childhood through adulthood. Most developmental studies have focused on white matter, often relying on diffusion–tensor imaging (DTI) models to recover FA, MD, radial diffusivity (RD), and axial diffusivity (AD) to quantify microstructural changes in pathways that support sensory, motor, cognitive, and affective functions. A comprehensive review of this literature is beyond the scope of the present manuscript, and several thorough summaries are already available (Lebel et al., 2019, Piekarski et al., 2023, Sousa et al., 2018, Goddings et al., 2021, Mah et al., 2017). Here, we provide a brief overview to contextualize the contributions of studies using ABCD data and to highlight the opportunities presented by future ABCD releases.

### 3.1 What did we know before ABCD: Typical development of white and grey matter microstructure

Synthesizing prior literature, Lebel et al. (2019) characterize white matter development as a prolonged, non-linear process extending from infancy through the third decade. FA generally increases and MD and RD decrease, while AD findings are inconsistent across tracts and studies, reflecting progressive organization and myelination (Fig. 6). Crucially, adolescence is not a self-contained epoch but a phase of continued, gradually slowing change. When analyzed in isolation, adolescent trajectories can appear noisy or contradictory (e.g., showing apparent “peaks”). However, when modeled over a wider age span (e.g., 5 to 30 years) using flexible functions, these curves are monotonic up to a tract-specific plateau, and apparent adolescent peaks or declines are largely artifacts of restricted age range and model choice.

At the same time, maturation exhibits substantial regional heterogeneity. Commissural and projection fibers appear largely mature by the end of adolescence, although timing varies within these classes. Callosal segments plateau relatively early, whereas the corticospinal tract continues to change into the twenties. In contrast, fronto-temporal association pathways, such as the superior longitudinal fasciculus, uncinate fasciculus, and cingulum, show protracted development well into the twenties. Consequently, adolescent changes are concentrated in high-order systems supporting complex cognition, emotion regulation, and executive function. This explains why adolescent samples show robust developmental effects in some tracts while appearing stable in others (Lebel et al., 2019, Goddings et al., 2021).

**Fig. 6:**
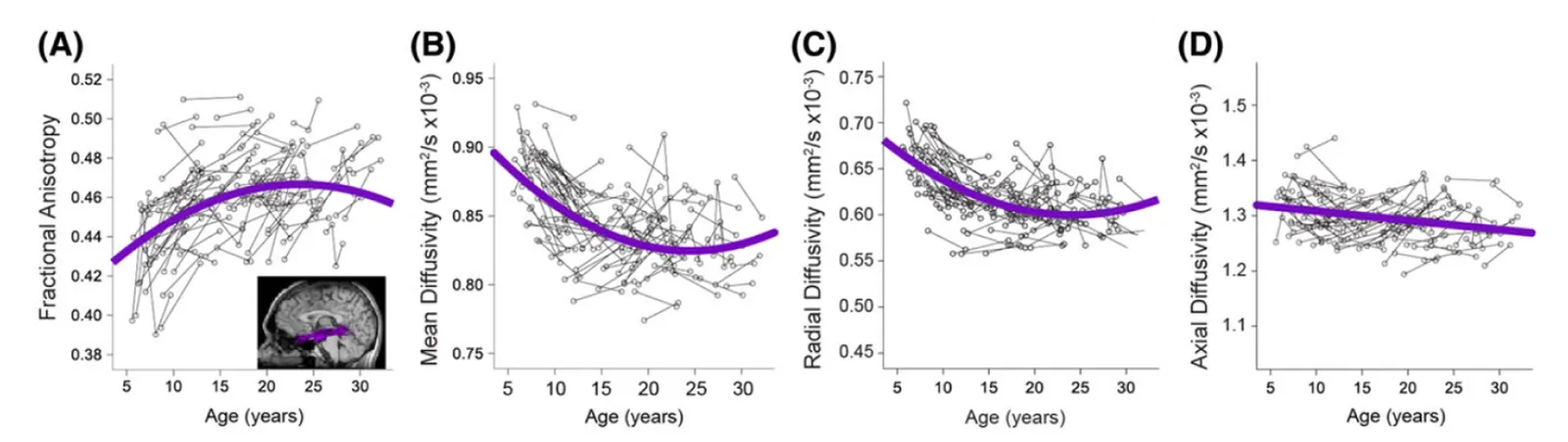
Example of diffusion parameters within a single tract (inferior longitudinal fasciculus, ILF) showing increases of fractional anisotropy (FA; A), decreases of mean diffusivity (MD; B), decreases of radial diffusivity (RD; C), and decreases of axial diffusivity (AD; D) with age in a longitudinal study from Lebel and Beaulieu (2011). Reproduced with permission from Lebel C, Treit S, Beaulieu C. (2019). A review of diffusion MRI of typical white matter development from early childhood to young adulthood. *NMR in Biomedicine, 32*:e3778.

Goddings et al. (2021) further review this protracted maturation, linking it to developing functions like inhibition and working memory. They note that cross-sectional correlations are often inconsistent, and that the small number of available longitudinal studies suggests earlier or faster microstructural development is associated with better inhibitory control and predicts later working memory capacity. Longitudinal data of this kind remain scarce across all executive functions. This work highlights methodologic challenges in pediatric imaging (e.g., motion, heterogeneous pipelines) and advocates for large population-based cohorts like ABCD, as well as multicompartment models such as Neurite Orientation Dispersion and Density Imaging (NODDI), whose parameters show closer associations with age in youth than standard DTI metrics. Thus, while DTI has dominated the field, models like NODDI clarify that developmental diffusion changes largely reflect increasing neurite density rather than geometric reorganization. For example, Mah et al. (2017) reported age-related increases in the neurite density index (NDI) in nearly all white matter tracts and subcortical gray matter structures in 8–13-year-olds, the caudate being the sole exception, while the orientation dispersion index (ODI) did not show significant age relationships anywhere. This indicates that reduced diffusivity tracks neurite packing or myelination rather than changes in fiber coherence. In cross-sectional developmental samples, NDI shows stronger age associations than FA (Genc et al., 2017, Mah et al., 2017, Chang et al., 2015), though this comparison carries the caveat that NODDI parameters are estimated from roughly twice as much diffusion data as the tensor metrics they are benchmarked against (Genc et al., 2017). Longitudinal and along-tract work converges on the same qualitative pattern of rising NDI alongside stable ODI (Geeraert et al., 2019, Lynch et al., 2020). These findings illustrate how multicompartment models can sharpen inferences about microstructural development, a motivation that heavily influenced the inclusion of RSI reconstruction in the ABCD protocol. However, the ABCD Study’s unprecedented sample size also offers a unique opportunity to stabilize and refine our understanding of traditional metrics. Thus, before detailing the novel contributions of multicompartment models, we first examine how this large-scale cohort has clarified white matter associations using standard DTI.

### 3.2 What we have learned from ABCD: Diffusion tensor imaging (DTI)

Recent studies have used ABCD’s massive sample size to resolve associations with DTI metrics that were previously inconsistent in smaller cohorts. For example, regarding typical demographic differences, Lawrence et al. (2023) identified widespread sex differences in white matter microstructure in over 6000 children. They found that boys generally exhibited higher MD, AD, and RD than girls, while FA differences were regionally specific (e.g., higher in boys in limbic tracts; higher in girls in association tracts). Against this typically developing backdrop, most ABCD DTI work has asked how these metrics relate to cognition, mental health, environment, and exposure, and we take each in turn below.

#### 3.2.1 Cognition and psychopathology

DTI metrics in ABCD have clarified the link between white matter indices, cognitive control, and mental health. Cardenas-Iniguez et al. (2022) demonstrated that higher executive function was associated with higher FA and lower MD across nearly all white matter tracts. Critically, while DTI metrics were not directly associated with general psychopathology or the specific internalizing factor, they were indirectly associated with conduct problems and ADHD via deficits in executive function. Other studies have pinpointed specific microstructural correlates for distinct phenotypes. Wiker et al. (2023) found that greater intra-individual variability in the slowest reaction times was specifically associated with an attention problems factor (*d* = 0.13–0.15) and with higher radial diffusivity in the bilateral corticospinal tract (*d* = 0.12 per hemisphere), with no comparable associations for other psychopathology dimensions. In contrast, Pagliaccio et al. (2021) reported that while obsessive-compulsive symptoms were associated with altered functional connectivity, only FA of the left corticospinal tract was associated with these symptoms, with a small effect size (β=−0.03), highlighting that extensive microstructural deficits may not be a universal feature of all psychopathology in this age group. Longitudinal analyses further suggest these associations may be elusive. Dall’Aglio et al. (2023) found no robust longitudinal coupling between global DTI metrics and broad internalizing or externalizing symptoms over two years, suggesting that white matter–symptom relationships may be stabilized earlier in childhood or require longer follow-up to detect change.

Genetic factors and methodological reliability also play a crucial role in interpreting these structural-behavioral links. Fernandez-Cabello et al. (2022) tested polygenic scores for six psychiatric disorders, a cross-disorder score, and educational attainment against imaging components spanning cortical and diffusion metrics in 7124 children. Bayes factors indicated null to anecdotal evidence for every psychiatric score. The clearest associations were for educational attainment, which related to larger global surface area, greater global white matter tract volume, and regional diffusion in cortico-striate pathways rather than to global FA or MD. Genetic liability for a cognitive trait was thus more readily detected in brain structure at this age than genetic liability for psychopathology, and even the authors’ multivariate models generalized poorly to held-out data. This suggests that genetic propensity for cognitive traits is partly instantiated in white matter microstructure, whereas psychopathology traits may be less so. Finally, Owens et al. (2023) investigated the test-retest reliability of neuroanatomical correlates of impulsivity over a 2-year interval. While they found stable associations between impulsivity and cortical surface area, associations with DTI metrics were less consistent, suggesting that microstructural biomarkers of personality traits may require larger effect sizes or more precise phenotyping to be reproducible longitudinally.

#### 3.2.2 Socioeconomic and physical health context

ABCD data have suggested that environmental and physical health factors may leave detectable imprints on white matter microstructure. Li et al. (2023a) showed that neighborhood disadvantage and lower parental education were independently associated with lower FA in a number of tracts, with the largest effects in the left superior longitudinal fasciculus. Notably, these effects were partially accounted for by cognitive performance, highlighting a pathway linking environmental adversity to neural development. Work by Thijssen and colleagues (Thijssen et al., 2020, Thijssen et al., 2022) has further linked stressful family environments to altered anterior cingulate FA, consistent with patterns of accelerated structural maturation observed in response to adversity. Specifically, their initial study Thijssen et al. (2020) found that the association between family environment and higher anterior cingulate FA was mediated by advanced pubertal development. While a subsequent replication study Thijssen et al. (2022) confirmed the direct link between family environment and FA, the mediation by pubertal stage was not replicated for this specific metric, suggesting a complex interplay of mechanisms.

Physical health and lifestyle also play major roles. For example, Kaltenhauser et al. (2023) reported that higher BMI and waist circumference were associated with pervasive reductions in FA, particularly in the corpus callosum. Longitudinally, higher baseline BMI predicted decelerated white matter development (smaller interval increases in FA) over two years, suggesting that metabolic health may impact the trajectory of microstructural maturation. Conversely, beneficial activities like sports participation show complex associations. Gorham and Barch (2020) found that greater involvement in sports was associated with reduced depressive symptoms in boys. However, contrary to the hypothesis that exercise improves white matter integrity, sports involvement was associated with lower FA in the fornix, potentially reflecting complex experience-dependent plasticity rather than simple “enhancement” of microstructure.

#### 3.2.3 Substance use and exposure

Finally, ABCD’s scale has enabled detection of subtle white matter alterations associated with early substance use and prenatal exposures, while also setting limits on what tensor metrics reveal about genetic liability. Sullivan et al. (2025) examined youth who initiated nicotine use between ages 9 and 16 and found that initiators exhibited “flattened” developmental trajectories of FA in a number of fiber pathways, with largest effects in corpus callosum, cingulum, and inferior longitudinal fasciculus compared to non-using peers, even after controlling for other substance exposures. Regarding prenatal risks, Long and Lebel (2022) found that even low levels of prenatal alcohol exposure (averaging one drink per week) were associated with lower FA in parietal, temporal, and occipital white matter, alongside higher externalizing symptoms. Similarly, Evanski et al. (2024) examined ten frontolimbic pathways and isolated the effect of prenatal cannabis exposure to the bilateral fornix, the only tracts to survive multiple comparison correction, though they emphasized that these effects were small (semipartial *r* = −0.03 to −0.04) and required large samples to detect reliably. In a matched subsample, Acosta-Rodriguez et al. (2025) reported a broader pattern spanning white matter tracts and cortical regions supporting goal-directed behavior.

Polygenic risk presents a different picture. Hatoum et al. (2021) tested polygenic scores for problematic alcohol use and drinks per week against cortical, subcortical, and white matter phenotypes in 3013 substance-naïve children. No FA or MD association survived correction; the surviving effects were lower left frontal pole grey matter volume (β=−0.057) and greater right supramarginal gyrus cortical thickness (β=0.032; both $p_{\mathrm{fdr}}<0.05$, $r^{2}<0.004$). Predispositional genetic risk for alcohol involvement is therefore detectable in brain structure before first use, but at this age it appears in morphometry rather than in tensor-derived microstructure. Together, these findings underscore that DTI remains a sensitive marker for the neurodevelopmental “footprints” of both prenatal and early-adolescent toxicant exposures, but questions about the meaningfulness of these effects remain (Dick et al., 2025).

#### 3.2.4 Summary

Together, these findings show that tensor metrics detect small but reproducible associations with developmental health once samples reach the thousands. The dominant pattern links higher FA and lower diffusivity to better cognitive control and to lower environmental adversity, obesity, and toxicant exposure. The pattern is not uniform, however. Greater family-related stress has been associated with higher regional FA, interpretable as accelerated maturation only under assumptions the tensor model cannot itself support. Replicability has likewise been uneven, with brain–behavior associations proving far less stable across a two-year interval than the imaging measures themselves. Both problems trace in part to the same source. Because FA and MD confound neurite density, crossing-fiber geometry, and extracellular free water, a change in either metric admits several incompatible biological readings, and the sign of an association carries little mechanistic information on its own. This limitation has motivated the shift toward multi-compartment models such as RSI and NODDI, already visible in several of the studies reviewed above and treated in detail in the next section.

### 3.3 What we have learned from ABCD: Restriction spectrum imaging (RSI) and other multicompartment models

The ABCD processing pipeline implements RSI, a multi-compartment diffusion model developed by Dale, White and colleagues (White et al., 2013a, White et al., 2013b, White et al., 2014, Brunsing et al., 2017), as its standard framework for characterizing tissue microstructure. Before turning to what RSI has revealed, it is worth addressing why the ABCD diffusion literature has centered on RSI rather than other multi-compartment models, most prominently NODDI (Zhang et al., 2012). At the level of the core microstructural index, the two models are more alike than different. Both decompose the diffusion signal into an intracellular (restricted) compartment, an extracellular (hindered) compartment, and a free-water compartment, and both yield a primary measure of intracellular density. NODDI’s intracellular volume fraction (ICVF, also termed the NDI) and RSI’s restricted signal fraction indices are dominated by restricted intracellular water which increases with age, putatively tracking largely the same maturational processes of neurite proliferation and myelination. Cross-cohort developmental studies bear this out for NODDI (Genc et al., 2017, Geeraert et al., 2019, Lynch et al., 2020, Mah et al., 2017), and recent ABCD work confirms that NODDI’s ICVF/NDI is among the most age-sensitive diffusion metrics available (Meisler et al., 2026).

The models differ in two respects. First, they parameterize the restricted signal differently. NODDI models fiber orientation dispersion through a Watson distribution and reports it as a dedicated index (ODI), whereas RSI partitions the restricted compartment into isotropic (RNI) and directional (RND) fractions (Palmer et al., 2022). Neither decomposition is richer; each simply foregrounds different features of the same data. Second, they handle orientation differently. NODDI represents neurites as sticks dispersed about a single dominant axis, which inflates ODI where fibers cross at large angles, whereas RSI recovers the restricted-compartment fiber orientation distribution by spherical deconvolution, modeling the restricted and hindered compartments as fourth-order spherical harmonics (Palmer et al., 2022, Zhang et al., 2012, White et al., 2013a). This is one reason RSI has been favored for the grey-matter and subcortical analyses that feature prominently in ABCD. Both models fix intrinsic compartmental diffusivities (in ABCD, RSI holds axial diffusivity at 1 × 10^−3^ mm^2^/s for the restricted and hindered compartments), and the default NODDI parallel diffusivity is known to be suboptimal in grey matter and in the infant brain (Palmer et al., 2022, Guerrero et al., 2019).

Given these similarities, RSI’s prominence in the ABCD literature reflects availability more than any demonstrated superiority. RSI metrics are computed in the standard ABCD pipeline (Hagler et al., 2019) and distributed in the curated tabulated releases, whereas NODDI, along with MAP-MRI and diffusion kurtosis metrics, are available through the separately curated ABCD-BIDS Community Collection. Both are now distributed via the NIH Brain Development Cohorts (NBDC) Data Hub (Meisler et al., 2026). To our knowledge, no study has yet compared RSI and NODDI head-to-head in ABCD, in part because the pipeline used to generate these derivatives does not currently support RSI reconstruction (Meisler et al., 2026). Therefore, a direct comparison of their developmental sensitivity in this cohort remains an open and worthwhile question.

The high-b shells (b≥3000) in ABCD’s multi-shell protocol are critical to this decomposition, as they suppress extracellular signal to isolate the signal from the intracellular space in both white and grey matter. Without this high-b sampling, the intracellular and extracellular pools would be poorly distinguished, and RSI would collapse back toward tensor-like behavior. Palmer et al. (2022) applied this framework to over 8000 9–14-year-olds. The study revealed developmental patterns that are largely invisible to standard DTI. They found that older participants showed higher restricted signal fractions globally (increases in RNT, RNI, RND) and concomitant decreases in hindered/free fractions (HNT, FNT). These shifts are consistent with maturational processes such as myelination and increased neurite packing, alongside reduction of extracellular space. As Palmer et al. (2022) emphasize (their Table 1), several distinct cellular changes, including increases in cell body size and microglial recruitment, produce the same directional change in the restricted fraction, so the diffusion signal alone does not adjudicate among them. Critically, while 9 to 14-year-olds are still on a clear ascending limb of microstructural maturation generally, the most striking effects appeared in subcortical grey matter rather than just in major white matter tracts (see Fig. 7). Specifically, RSI effects were significantly larger and more spatially specific in the basal ganglia (globus pallidus, putamen, ventral striatum, ventral pallidum), thalamic nuclei, ventral diencephalon, and regions around the substantia nigra/ventral tegmental area.

Relative to the earlier DTI literature synthesized by Lebel and others, these findings refine our picture of adolescent brain development in several ways. First, the robust changes in the ventral forebrain and midbrain point to ongoing refinement of dopaminergic and fronto-striatal-thalamic circuitry rather than a simple “plateau” in adolescence. Second, voxelwise RSI analyses reveal marked heterogeneity within structures and along tracts that would be obscured in ROI-based DTI work. For example, different thalamic nuclei show distinct age-RSI profiles and within-tract hotspots of age-related change cluster near “innervation zones” where tracts interface with subcortical nuclei. Three constraints on this evidence are worth noting in a methodological review. The voxelwise effects, although extraordinarily significant, are small in magnitude, which the authors attribute to the realistic effect sizes obtainable in samples of this size. The analysis used two timepoints, so regional differences in the shape of developmental trajectories could not be separated. And because imaging metrics were rank-normalized to satisfy linear model assumptions, nonlinear age effects were not modeled.

**Fig. 7:**
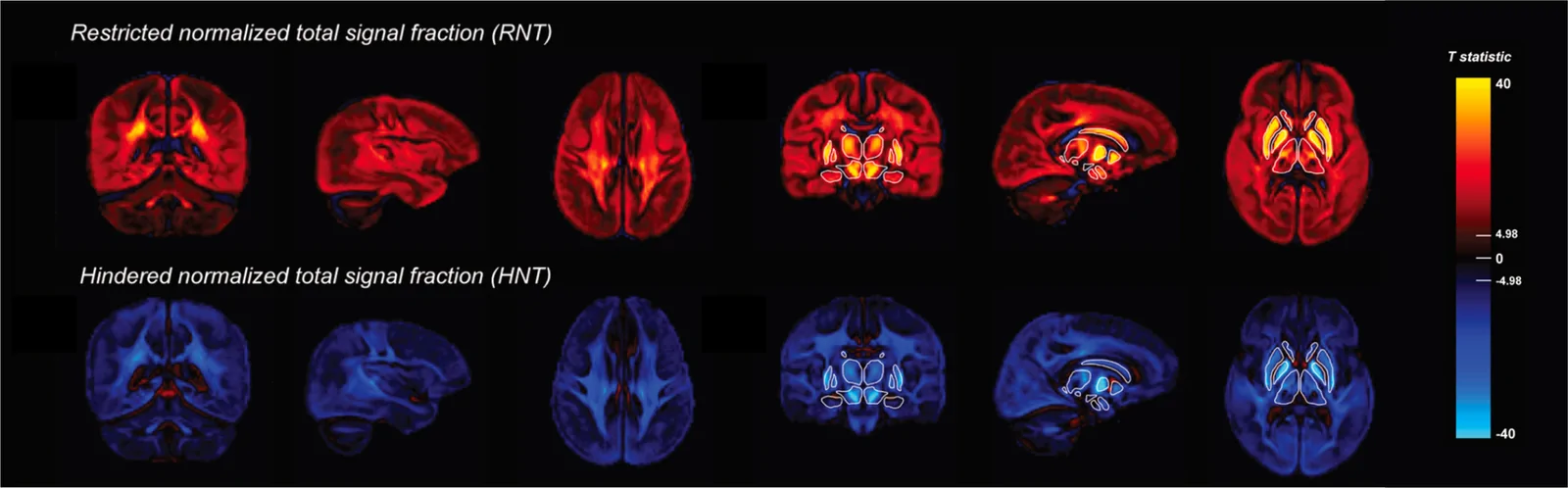
Voxelwise associations between restricted and hindered diffusion and age, controlling for covariates. Adapted from Palmer, C. E., Pecheva, D., Iversen, J. R., Hagler, D. J., Jr., Sugrue, L., Nedelec, P., Fan, C. C., Thompson, W. K., Jernigan, T. L., and Dale, A. M. (2022). Microstructural development from 9 to 14 years: Evidence from the ABCD Study. *Developmental Cognitive Neuroscience, 53*, 101044, under a CC BY 4.0 license.

This sharpened view of typical microstructure has provided a sensitive baseline for interrogating how environmental factors shape brain development at the cellular level (Conley et al., 2021). In baseline cross-sectional work, RSI metrics have revealed that relatively low levels of outdoor air pollution are associated with measurable differences in intracellular composition (Burnor et al., 2021, Sukumaran et al., 2023). For example, annual PM_2.5_ exposure at ages 9–10 was linked to higher RNI in fronto-limbic association tracts (Burnor et al., 2021) and higher subcortical RNI in the thalamus, brainstem, and nucleus accumbens (Sukumaran et al., 2023). Sukumaran et al. (2023) further reported that higher RNI in these regions was linked to better cognitive test performance and to greater internalizing but fewer externalizing problems, a pattern whose interpretation is not straightforward but which indicates these microstructural differences are not behaviorally inert at exposure levels below current U.S. standards. Complementary work using structural rather than diffusion metrics links these same pollutants to altered cortical thickness trajectories across adolescence (Jara et al., 2026).

Longitudinal work extends this picture, showing that pollutants can alter the trajectory of maturation. Cotter et al. (2024) found that PM_2.5_ was associated with higher attained RND at age 9 in both sexes but did not predict change over the following two years, whereas NO_2_ and O_3_ were associated with altered rates of change in RNI and RND, with the affected tracts differing by pollutant and sex. Moving to source-specific exposures, Bottenhorn et al. (2024) used longitudinal partial least squares modeling to show that PM_2.5_ specifically from biomass burning was selectively associated with deviations in cortical neurite development. Together, these studies illustrate how RSI’s compartment-resolved measures can pinpoint which pollution sources disrupt neurite and cellular development, providing a mechanistic bridge between environmental epidemiology and neuroimaging.

RSI has also clarified how obesity and early weight gain relate to the microstructure of reward circuitry. Rapuano et al. (2020) showed that greater waist circumference was associated with higher restricted isotropic diffusion in the nucleus accumbens, consistent with increased cellularity.^1^ These “hot spots” of increased cellularity also predicted weight gain over the following year, with spatial specificity to the ventral striatum (see Fig. 8). Extending the interval, Rapuano et al. (2022) found that this prediction held over two years and that baseline weight predicted subsequent increases in nucleus accumbens cellularity. This bidirectional relationship is speculatively offered as evidence of a self-reinforcing cycle of neuroinflammation and weight gain. Adise et al. (2025) broadened this finding, showing that while RNI tracks age in healthy-weight youth, it tracks BMI in those gaining excess weight. Two further studies provide convergent validity. Bayat et al. (2026) replicated this general pattern using the RSI ABCD acquisition and reconstruction in a younger sample of 4–7-year-olds, extending the finding below the ABCD age range. And using a different model, Diffusion Basis Spectrum Imaging (DBSI), Li et al. (2023b) replicated the striatal associations in ABCD while further localizing obesity-linked cellularity increases to the hypothalamus and widespread white matter.

Finally, recent twin analyses demonstrate that the microstructural signals most altered by these environmental exposures are also those under strong genetic influence. Watts et al. (2023) estimated genetic and environmental contributions to RSI metrics in ABCD’s twin sample, finding that subcortical RNI is far more heritable (>0.80 in pallidum, putamen, and thalamus) than traditional DTI metrics. Longitudinally, RNI genetic correlations were high across all nine regions ($r_{A}$ = 0.74 in the amygdala to 0.97 in the caudate), indicating that the genetic architecture of deep grey matter microstructure is largely conserved across early adolescence. Outside the hippocampus and caudate these correlations fell short of 1.0, implying some new genetic variance over the interval, though the magnitudes are high enough that the picture is one of mostly stable genetic influence rather than substantial remodeling. Together with its cross-time stability, the heritability of RNI positions it as a strong candidate intermediate phenotype for imaging-genetics studies aimed at parsing how genetic risk and environmental exposure interact to shape subcortical maturation.

**Fig. 8:**
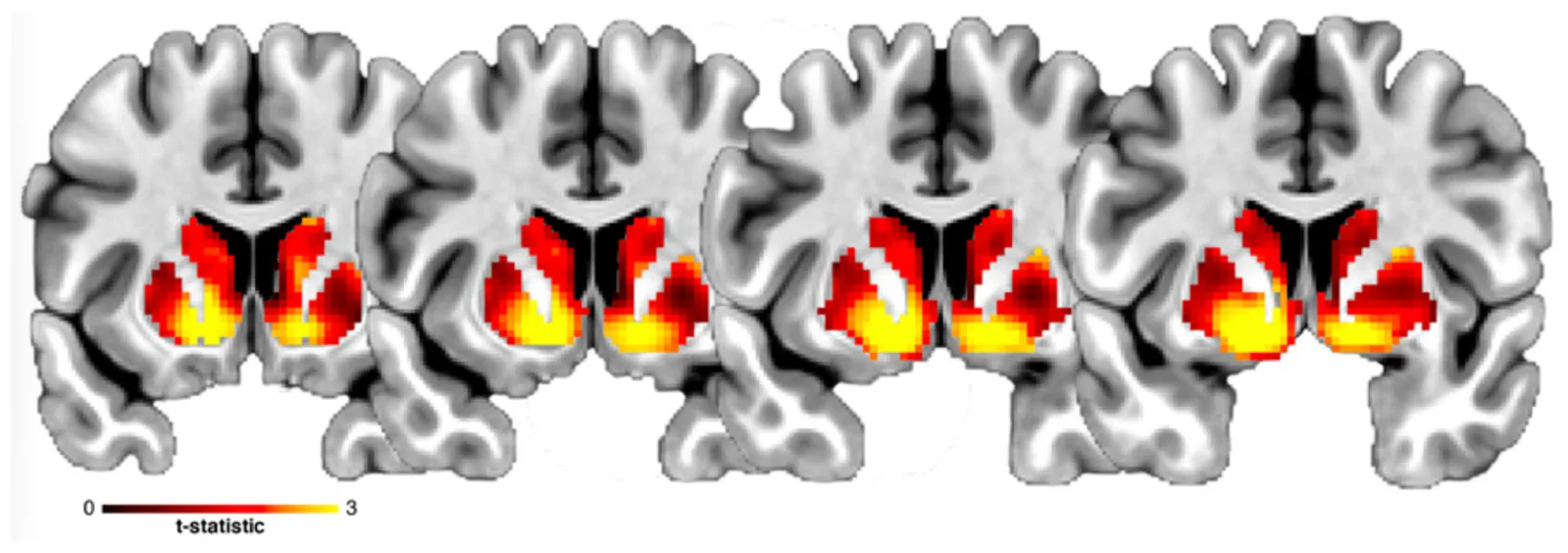
Voxelwise prediction of 1-y change in waist circumference from restricted diffusion fraction (accounting for covariates including baseline waist circumference). Higher *t*-values (yellow) demonstrate spatial specificity in the ventral striatum. Reprinted with permission from Rapuano, K. M., Laurent, J. S., Hagler, D. J., Jr., Hatton, S. N., Thompson, W. K., Jernigan, T. L., Dale, A. M., Casey, B. J., and Watts, R. (2020). Nucleus accumbens cytoarchitecture predicts weight gain in children. *Proceedings of the National Academy of Sciences, 117*, 26977-26984.

### 3.4 Integrating structural and diffusion perspectives on adolescent maturation

Having reviewed structural morphometry and diffusion microstructure separately, it is worth asking where the two modalities converge and where they index distinct biology. The distinction matters because a review organized by modality can leave the impression that CT, SA, and RSI metrics are interchangeable windows onto “maturation”, when in fact their complementarity is the reason ABCD collects both.

Several lines of convergence are apparent. First, both localize the most protracted change to overlapping circuitry rather than to uniform maturation. Cortical surface area shows a broadly inverted-U trajectory, peaking around age 11 to 12, with parietal and much of occipital cortex among the earliest regions to turn over into decline and medial frontal and temporal cortex showing the most protracted increases (Smith et al., 2025). Consistent with this, Marshall et al. (2025), examining ages 9 to 13, found parietotemporal decreases alongside frontotemporal increases over that interval; medial-occipital regions, however, were still increasing in their sample, suggesting occipital cortex is not uniformly early-peaking and that some subregions may turn over later than the broader occipital pattern implies. RSI restricted-signal increases are largest in the basal ganglia, thalamic nuclei, and ventral diencephalon, and within white matter cluster where tracts innervate subcortical structures (Palmer et al., 2022). These results implicate fronto-striatal-thalamic systems as among the latest-maturing, although there is marked heterogeneity within conventional parcels. Second, both exhibit the same brain–behavior signature. That is, the signature is one of reliable but small, spatially distributed associations requiring large samples for stable detection, whether the phenotype is cognition, psychopathology, or substance-use initiation (Marek et al., 2022, Miller et al., 2024). Third, both are vulnerable to the same confounds (global brain size, sociodemographic context, and in-scanner motion), so the covariate considerations in Section 1.3 apply to both. Finally, both are substantially heritable, and the genetic signals appear partly distinct. Subcortical RSI microstructure is among the most heritable imaging phenotypes reported in ABCD twin analyses, exceeding tensor-derived diffusivity in the same regions (Watts et al., 2023), while cortical thickness and surface area carry their own strong genetic contributions (Smith et al., 2024). The heritable variance in each case is anchored to anatomy the other modality characterizes poorly, and both signals are stable across the two-year interval separating ABCD imaging waves.

The divergences are what make the modalities complementary rather than redundant. Structural morphometry is intrinsically macroscopic. That is, CT and SA quantify the geometry of the cortical sheet but cannot distinguish the cellular processes (synaptic pruning, myelination near the mantle, dendritic change) that produce it. Apparent thinning can even reflect increasing intracortical myelin shifting the grey–white boundary rather than tissue loss (Natu et al., 2019, Lorio et al., 2016). RSI and related multi-compartment models were designed to address this ambiguity, resolving restricted, hindered, and free-water compartments that are sensitive to, though not specific to, neurite and cell-body density (Palmer et al., 2022). The two modalities are also informative about different anatomy. sMRI volumetrics say little about the internal microstructure of the basal ganglia and thalamus, which is exactly where RSI shows its most distinctive age effects, and diffusion imaging additionally characterizes white-matter pathways that morphometry indexes only as volume. A converging account therefore emerges only when the modalities are read together, and the mechanistic interpretation of any single structural change frequently depends on microstructural evidence that sMRI alone cannot supply.

This complementarity motivates the multimodal, methodologically unified agenda we turn to next. The obstacle has been that structural, diffusion, and genetic data have typically been modeled in separate pipelines at incompatible scales. The innovations reviewed next, harmonized multimodal registration and mixed-effects modeling at whole-brain resolution, are what make integrated developmental inference tractable at ABCD scale.

## 4 Innovations and methodological challenges

The ABCD data set comes with two major challenges that necessitated a substantive advance in the tools available for analyzing imaging data. The first challenge is the size of the dataset, and the second is the longitudinal structure. The longitudinal structure requires handling both a larger number of scans, which will continue to propagate over the term of the project, and appropriate modeling of the covariance structure of longitudinal data. FEMA was developed to deal with both issues, making it possible to analyze whole-brain vertex-, voxel-, and connectome-wise data quickly, and through web interfaces developed by the ABCD Data Exploration and Analysis Portal (DEAP).

### 4.1 FEMA for large-scale structural imaging: Development and applications

Efficiently modeling complex dependence structures (site, family, subject, twinship) at whole-brain resolution has historically been prohibitive for large cohorts. To address this, FEMA was developed to make vertex-, voxel-, and connectome-wise linear mixed-effects feasible at ABCD scale. A necessary prerequisite was the creation of the Multimodal Image Normalisation Tool (MINT; Pecheva et al., 2022). MINT generates a cohort-specific atlas from 19,000 scans using eleven imaging channels, including structural MRI and diffusion-derived spherical harmonics. By leveraging orientational information, MINT achieves precise alignment of white-matter pathways and subcortical grey matter at a fraction of the computational cost of traditional tools, providing the foundational voxelwise correspondence required for mass-univariate modeling.

The FEMA framework itself employs a three-step statistical approach to maximize computational efficiency: (1) an initial ordinary least squares (OLS) step for estimation of the fixed effects, (2) a method-of-moments (or regression) estimator using squares and products of OLS residuals to obtain variance components under non-negativity constraints, and (3) a generalized least squares update for the fixed effects coefficients. By exploiting sparsity in random-effects design matrices, FEMA achieves orders-of-magnitude speed gains over standard solvers while maintaining statistical equivalence to maximum likelihood estimates. Parekh et al. (2024) validated this approach in ABCD Release 4.0, demonstrating accurate recovery of longitudinal age effects on cortical thickness and functional connectivity at full resolution.

FEMA has subsequently enabled granular genetic modeling that was previously computationally intractable. Smith et al. (2023) extended standard twin designs by incorporating genome-wide SNP-relatedness into FEMA models (n=9742), tightening heritability estimates for cognitive traits. Subsequent vertex-wise analyses partitioned cortical variance into genetic, shared-environmental, and stable person-specific components. These maps revealed significant heterogeneity within classical ROIs. For example, the supramarginal gyrus contains subzones with distinct genetic versus subject-specific signatures, highlighting the necessity of vertex-level resolution (Smith et al., 2024).

Recently, FEMA-Long has been introduced, which extends the FEMA framework to include modeling using splines, time-varying random effects using unstructured covariance, and additional scalability for performing genome-wide association analyses to discover genetic variants that show time-dependent effects (Parekh et al., 2026). Smith et al. (2025) used FEMA with spline modeling for ABCD Release 6.0, to estimate smooth, vertex-wise developmental curves for thickness, surface area, and local expansion (log-Jacobian) while rigorously adjusting for longitudinal nesting and scanner effects.

Finally, FEMA has been integrated into scalable toolkits and applied to substantive behavioral questions. Im et al. (2025) packaged FEMA for subcortical surface analysis, reducing runtimes by ∼16× relative to standard pipelines. In application, Rauschecker et al. (2025) used these tools to disentangle the neural correlates of media consumption, identifying regionally selective associations between reading and increased cortical area that were robust to family and site confounds.

FEMA has also been integrated into the DEAP, a web-based platform that supports the full research lifecycle, from data exploration to hypothesis-driven analysis and results visualization. Within DEAP’s analysis module, FEMA enables complete specification of linear mixed-effects models through a graphical user interface, including covariates, random effects, covariance structures, fixed-effect transformations (e.g., mean centering or creation of splines), and *n*-way interactions. Together, these tools surmount the computational barriers of large-scale longitudinal imaging, establishing a unified framework for reproducible, voxel- and vertex-wise inference across the developing brain.

## 5 Toward integration

Across the domains reviewed in this paper, a central theme emerges: large-scale, harmonized, multimodal datasets have fundamentally reshaped what can be inferred about structural brain development and its relation to behavior. Early work in developmental neuroimaging was limited by small samples, heterogeneous preprocessing pipelines, and insufficient power to adjudicate among competing developmental models. The ABCD Study, with its scope and longitudinal depth, has provided a framework for revisiting these questions with vastly improved precision. Structural MRI now offers more stable and reproducible estimates of cortical and subcortical development, while diffusion models, including RSI and other multi-compartment approaches, have revealed distinct microstructural trajectories that were previously masked by measurement noise and inadequate sample sizes.

Instead of coarse or contradictory developmental patterns, we now see differentiated and replicable trends that align more closely with specific biological hypotheses. For example, once motion and scaling are handled rigorously, cortical thickness shows predominantly linear thinning between ABCD waves, which is more consistent with gradual refinement than with a mid-adolescent structural “inflection” as the dominant account of thickness maturation. Surface area is a different case. Earlier work describes an early-adolescent SA peak (Section 2.1.3), while two-wave ABCD data show regionally mixed expansion and contraction (Section 2.2), with the shift from expansion to contraction appearing later in males in some regions (Section 2.2.1). Whether SA has a common mid-adolescent turning point therefore remains open. Likewise, the concentration of RSI restricted-diffusion increases in basal ganglia, thalamus, and ventral midbrain points to ongoing maturation of dopaminergic and fronto-striatal-thalamic circuitry during this window. And the high heritability of subcortical microstructure relative to traditional diffusion metrics identifies deep grey matter as a genetically constrained target for imaging-genetics work. Each of these is a concrete, testable claim that small-sample, single-modality studies could not previously adjudicate.

ABCD has also clarified long-standing ambiguities in brain–behavior associations. Findings that once appeared robust in small samples, such as strong correlations between CT and cognition, or large group differences in psychopathology, have consistently diminished in magnitude when examined at larger scales. This reframing is not a limitation but a methodological advance: effect sizes are now better estimated, confidence intervals are narrower, and researchers are less likely to be misled by sampling variability or publication bias. The study has also illuminated the substantial influence of covariates like sex, age, motion, scanner manufacturer, and socioeconomic context that earlier studies could not address simultaneously. At the same time, the longitudinal design of ABCD has begun to reveal developmental signals, including approximately linear cortical thinning within the early-adolescent window (though two waves cannot establish trajectory shape), stable person-specific variance in cortical and subcortical structure that does not appear to be explained by heritability alone, and regional specificity in brain–behavior relationships that appears to strengthen across the ages sampled. With additional waves, spline-based whole-brain models are now beginning to estimate trajectory shape directly rather than slope alone (Smith et al., 2025).

Interpretation nonetheless remains bounded by what current measures and designs can resolve, and it is worth stating these limits plainly. Macrostructural change is still mechanistically ambiguous. For example, a given reduction in cortical thickness may reflect synaptic pruning, increased intracortical myelination shifting the grey-white boundary, or partial-volume and contrast effects, and morphometry alone cannot adjudicate among them (Natu et al., 2019, Lorio et al., 2016). Microstructural models narrow this ambiguity but do not eliminate it. Restricted-diffusion signal is sensitive to intracellular density, but does not by itself distinguish neurite proliferation from glial or cell-body changes, and competing multi-compartment models have not been benchmarked head-to-head in ABCD (Palmer et al., 2022, Meisler et al., 2026). Design constraints compound these measurement limits. Most ABCD structural findings still rest on two waves, which can localize the timing of an inflection but cannot yet characterize the shape of individual trajectories or firmly separate maturational change from exposure effects. Furthermore, because acquisition upgrades are partly confounded with participant age, some apparent developmental effects remain entangled with scanner and software transitions, and it is important to resolve this in future analyses with more data collection waves. Recognizing these boundaries is not a counsel of caution so much as a specification of what the next waves, and genuinely multimodal models, are positioned to resolve.

Looking forward, the next stage of integration will rely on models that treat the brain as a coordinated, multi-level system rather than a set of isolated measurements. Methodological advances developed within ABCD, such as MINT for harmonized multimodal registration, FEMA for scalable voxelwise mixed-effects modeling, and improved pipelines for motion quantification, missing-data handling, and effect-size benchmarking, now make it possible to evaluate structural, diffusion, functional, and genetic information within unified longitudinal frameworks. These tools allow researchers to decompose developmental change into global, regional, and network-level components, to quantify stability and reorganization across time, and to identify when and where structural metrics provide incremental value beyond demographics, environment, or genetics. Integrating across modalities will be essential for disentangling whether observed structural differences reflect cellular composition (myelination, neurite density, inflammation), morphometric reorganization, or developmental timing shifts.

As ABCD participants transition into young adulthood, opportunities for deeper integration will only grow. The move into adulthood will allow researchers to examine the persistence or attenuation of adolescent brain signatures, the emergence of new structural–behavior associations, and the interaction between early neurodevelopmental trajectories and adult outcomes in cognition, mental health, substance use, and physical health. Specifically, longer time intervals will enable identification of developmental inflection points, or periods when structural features become more predictive of individual differences or more sensitive to environmental exposures. With continued methodological refinement and increasingly rich multimodal data, the field is poised to transition from mapping typical developmental trends to understanding how heterogeneous pathways unfold, where they diverge, and how they may ultimately inform prevention, intervention, and policy.

## CRediT authorship contribution statement

**Anthony Steven Dick:** Writing – original draft, Formal analysis. **Donald J. Hagler Jr.:** Writing – review & editing. **Alison Rigby:** Writing – review & editing. **Mohammadreza Bayat:** Writing – review & editing. **Marilyn Curtis:** Writing – review & editing. **Raul Gonzalez:** Writing – review & editing. **Carolina Makowski:** Writing – review & editing. **Diliana Pecheva:** Writing – review & editing. **Alexander L. Wallace:** Writing – review & editing. **Richard Watts:** Writing – review & editing. **Biplabendu Das:** Data curation. **Janosch Linkersdoerfer:** Data curation. **Olivier Celhay:** Data curation. **Pravesh Parekh:** Writing – review & editing. **Eva Müller-Oehring:** Writing – review & editing. **Mustafa Okan Irfanoglu:** Writing – review & editing. **Elizabeth A. Hoffman:** Project administration, Writing – review & editing. **Gayathri J. Dowling:** Project administration, Writing – review & editing. **Diana Alkire:** Data curation. **Anders Dale:** Writing – review & editing. **Terry Jernigan:** Writing – review & editing.

## Declaration of Generative AI and AI-assisted technologies in the writing process

During the preparation of this work the authors used Anthropic Claude, OpenAI ChatGPT, and Google Gemini to facilitate R coding, to check grammar and flow of content originally drafted by the authors, to check reported findings and effect sizes against source publications, and to suggest revisions to draft text. All substantive claims, citations, numerical values, and interpretations were verified by the authors against primary sources. After using these tools, the authors reviewed and edited the content as needed and take full responsibility for the content of the publication.

## Declaration of competing interest

The authors declare the following financial interests/personal relationships which may be considered as potential competing interests: All authors report financial support was provided by the National Institutes of Health, and the National Institute on Drug Abuse. Anders Dale is a Founding Director of, and holds equity in, CorTechs Labs, Inc. (DBA Cortechs.ai), Precision Pro, Inc., Precision Health AS, Precision Health and Wellness, Inc., and Diploid Genomics, Inc. His relationships with these companies include board membership, consulting or advisory roles, and equity. All other authors declare that they have no known competing financial interests or personal relationships that could have appeared to influence the work reported in this paper.

## Appendix A Supplementary data

MMC S1

## Footnotes

1 Here and elsewhere, we use “cellularity” in the sense of higher intracellular restricted signal fraction, which may reflect gliosis, increased glial or neuronal cell bodies, or related processes.
