# Supplementary material for: Mapping the structure and microstructure of the adolescent brain: Insights from the ABCD study
Source: Dev Cogn Neurosci. 2026 Oct 30;82:101843. doi: 10.1016/j.dcn.2026.101843 (PMC13762968; doi:10.1016/j.dcn.2026.101843)
Supplement: Supplementary file 1 [file mmc1.pdf]

Supplemental Table 1: Completion rates and scan counts [% (*N*)] for T1, T2, and dMRI at each Timepoint by Demographic.

	T1				T2				dMRI			
Demographic	Baseline	2-Yr	4-Yr	6-Yr	Baseline	2-Yr	4-Yr	6-Yr	Baseline	2-Yr	4-Yr	6-Yr
Sex												
Male	99.3% (6,145)	70.7% (4,377)	55.1% (3,412)	34.7% (2,149)	95.2% (5,892)	69.1% (4,278)	54.2% (3,354)	34.3% (2,124)	94.6% (5,857)	69.0% (4,274)	54.0% (3,344)	34.2% (2,116)
Female	99.2% (5,630)	65.9% (3,740)	51.9% (2,949)	34.3% (1,945)	96.3% (5,469)	64.9% (3,683)	51.2% (2,906)	33.9% (1,926)	95.6% (5,431)	64.7% (3,674)	51.0% (2,897)	33.8% (1,921)
Ethnicity/Race												
Hispanic	99.4% (1,188)	68.0% (813)	54.9% (656)	33.3% (398)	94.9% (1,134)	66.9% (799)	53.9% (644)	32.8% (392)	94.4% (1,128)	66.4% (794)	53.9% (644)	32.6% (390)
White	99.5% (2,432)	64.6% (1,579)	51.6% (1,261)	31.4% (768)	95.9% (2,345)	63.4% (1,551)	50.7% (1,240)	31.1% (761)	95.1% (2,325)	63.2% (1,545)	50.3% (1,229)	30.9% (756)
Black	99.6% (228)	58.5% (134)	50.2% (115)	29.3% (67)	96.5% (221)	56.8% (130)	49.8% (114)	29.3% (67)	95.2% (218)	55.9% (128)	49.3% (113)	29.3% (67)
Asian	100.0% (2)	0.0% (0)	0.0% (0)	0.0% (0)	100.0% (2)	0.0% (0)	0.0% (0)	0.0% (0)	100.0% (2)	0.0% (0)	0.0% (0)	0.0% (0)
Other	99.3% (6,143)	72.2% (4,463)	54.6% (3,380)	39.0% (2,415)	96.7% (5,982)	71.1% (4,398)	54.0% (3,338)	38.7% (2,394)	96.1% (5,942)	71.0% (4,390)	53.8% (3,329)	38.6% (2,386)
Unknown	98.3% (1,782)	62.3% (1,128)	52.4% (949)	24.6% (446)	92.5% (1,677)	59.8% (1,083)	51.0% (924)	24.1% (436)	92.3% (1,673)	60.2% (1,091)	51.1% (926)	24.2% (438)
Household Income												
< $50k	99.0% (3,189)	66.0% (2,126)	52.2% (1,683)	30.6% (985)	94.0% (3,029)	64.2% (2,068)	51.1% (1,645)	30.2% (974)	93.3% (3,005)	64.1% (2,066)	51.0% (1,644)	30.2% (972)
$50k–$100k	99.3% (3,047)	72.8% (2,232)	56.3% (1,726)	37.4% (1,146)	95.6% (2,933)	71.5% (2,195)	55.4% (1,700)	36.8% (1,130)	95.0% (2,914)	71.4% (2,192)	55.1% (1,691)	36.8% (1,128)
> $100k	99.4% (4,533)	68.6% (3,130)	54.0% (2,465)	37.2% (1,698)	97.3% (4,440)	67.6% (3,081)	53.3% (2,433)	36.9% (1,684)	96.7% (4,411)	67.5% (3,077)	53.2% (2,428)	36.7% (1,676)
Unknown	98.9% (1,006)	61.8% (629)	47.9% (487)	26.1% (265)	94.3% (959)	60.7% (617)	47.4% (482)	25.8% (262)	94.2% (958)	60.3% (613)	47.0% (478)	25.7% (261)

Note. Year 6 only contains about half of the projected participants, so sample sizes at this time-point do not reflect full completion rates. Additional scans will be available in future data releases for the 6-Yr follow-up.
